# Primate social attention: Species differences and effects of individual experience in humans, great apes, and macaques

**DOI:** 10.1371/journal.pone.0193283

**Published:** 2018-02-23

**Authors:** Fumihiro Kano, Stephen V. Shepherd, Satoshi Hirata, Josep Call

**Affiliations:** 1 Kumamoto Sanctuary, Wildlife Research Center, Kyoto University, Kumamoto, Japan; 2 The Rockefeller University, New York, New York, United States of America; 3 Department of Developmental and Comparative Psychology, Max-Planck Institute for Evolutionary Anthropology, Leipzig, Germany; 4 School of Psychology and Neuroscience, University of St Andrews, St Andrews, United Kingdom; Centre de neuroscience cognitive, FRANCE

## Abstract

When viewing social scenes, humans and nonhuman primates focus on particular features, such as the models’ eyes, mouth, and action targets. Previous studies reported that such viewing patterns vary significantly across individuals in humans, and also across closely-related primate species. However, the nature of these individual and species differences remains unclear, particularly among nonhuman primates. In large samples of human and nonhuman primates, we examined species differences and the effects of experience on patterns of gaze toward social movies. Experiment 1 examined the species differences across rhesus macaques, nonhuman apes (bonobos, chimpanzees, and orangutans), and humans while they viewed movies of various animals’ species-typical behaviors. We found that each species had distinct viewing patterns of the models’ faces, eyes, mouths, and action targets. Experiment 2 tested the effect of individuals’ experience on chimpanzee and human viewing patterns. We presented movies depicting natural behaviors of chimpanzees to three groups of chimpanzees (individuals from a zoo, a sanctuary, and a research institute) differing in their early social and physical experiences. We also presented the same movies to human adults and children differing in their expertise with chimpanzees (experts vs. novices) or movie-viewing generally (adults vs. preschoolers). Individuals varied within each species in their patterns of gaze toward models’ faces, eyes, mouths, and action targets depending on their unique individual experiences. We thus found that the viewing patterns for social stimuli are both individual- and species-specific in these closely-related primates. Such individual/species-specificities are likely related to both individual experience and species-typical temperament, suggesting that primate individuals acquire their unique attentional biases through both ontogeny and evolution. Such unique attentional biases may help them learn efficiently about their particular social environments.

## Introduction

Human and nonhuman primates attend to other individuals to gain valuable social information about them (such as identity and emotions) and their shared surroundings (such as nearby dangers and resources), and even to infer others’ goals and intentions based on their actions. Fundamental characteristics of social attention are similar between human and nonhuman primates [1–3]. Primates selectively attend to others’ faces, eyes, and targets of ongoing actions [4–7]. They follow others’ gaze and attend to the same objects and locations that others are manipulating [8–12]. They anticipatorily attend to the targets of others’ actions before their actions are completed; for example, apes and macaques look at the goal objects while the actor is reaching to the object, before the actor grabs them [13–15]. Previous studies also suggested that human and nonhuman primates share common neurophysiological mechanisms underlying social information processing and that they process social information mainly through the two distinct pathways in their brains [2, 9, 16, 17]. One pathway, via subcortical routes, rapidly processes crude social information such as others' faces, eyes, and gaze direction; the other pathway, via cortical routes, processes nuanced social information such as others’ social and emotional status and communicative intentions [2, 9, 16, 17].

Another important feature of social attention is its individual variation. On the one hand, such individual variation in social orienting is related to biological, early-developing, temperamental characteristics in human and nonhuman primates. Attention to others’ eyes is evident at a very young age in human and nonhuman infants [18, 19]. Attention to targets of others’ gaze, pointing, and manual actions ("joint attention") also emerges early in the development (although somewhat later than attention to the eyes [20–23]). However, human children diagnosed with autism spectrum disorder attend to others’ eyes and targets of gaze and pointing less strongly than do typically-developing children [24–27]; even preverbal human infants later diagnosed with autism attend to eyes less strongly than typically-developing infants during viewing of social movies [26]. A recent study showed that monozygotic-twin infants show more similar levels of attention to the eyes than dizygotic-twin infants during viewing of social movies [27]. It is also known that endocrine systems mediate social attention in human and nonhuman primates: Human yearlings who experienced higher levels of prenatal androgen show lower levels of eye contact with their mothers [28], and oxytocin administration leads humans and monkeys to increase the levels of eye contact with the conspecific images [29, 30].

On the other hand, individual variation in social orienting is related to late-developing, experience-dependent characteristics in human and nonhuman primates. For example, human (sighted) infants of blind parents attend less to the eyes and gaze direction of parents compared to control infants [31, 32]. “Enculturated” apes, reared by humans in the human cultural environment, respond more than non-enculturated apes to the targets of human experimenters’ gaze, pointing, and manual actions when interacting with the experimenters [33–35]. In humans, it is known that cultural background biases attention to both social and physical stimuli. People from East Asian countries tend to attend to the central parts of faces (i.e., around the nose), while people from Western countries tend to directly attend to both eyes and mouth [36]. These two cultural groups differ in the same way even when presented with allospecific faces (e.g., a sheep face) and visually homogeneous non-face objects [37]. It is also known that expertise by profession biases attention among both social and physical stimuli [38–40].

Patterns of social orienting differ not only across individuals within a species but also across closely-related primate species. Previous studies used eye-tracking to compare social orienting between different primate species: macaques and humans [5, 41, 42], chimpanzees and humans [43], orangutans, gorillas and humans [44], and bonobos and chimpanzees [4]. Great apes and macaques, like humans, view the models’ faces and especially the eyes when presented with still pictures and movies [4–7, 43–48]. Also, like humans, apes and macaques view the targets of models’ manual actions in movies—even anticipatorily looking at targets of their manual actions [14, 49, 50]. However, compared to humans, apes and macaques view the targets of models’ actions for a shorter time during viewing of movies [5, 15]. Compared to humans, apes view the models’ eyes for a shorter time and the models’ mouth for a longer time when presented with pictures [43, 44]. It was also reported that when chimpanzees and bonobos view pictures, bonobos look at the model’s eyes for a longer time and the targets of models’ actions for a shorter time than do chimpanzees [4]. Therefore, although all these primate species view the same social features in pictures and movies—other’s faces, eyes, mouths, and action targets—they differ significantly from one another in the relative strength of viewing of each social feature. Such species-typical viewing patterns likely reflect temperamental characteristics unique to each species.

These previous studies established a useful paradigm for comparing viewing patterns of social stimuli across individuals and species under the same experimental conditions. However, the obtained results are still fragmentary with regard to the pattern and nature of the individual and species variations, particularly among nonhuman primates. More specifically, most previous studies compared only two species, and thus procedural differences across studies, such as the differences in stimuli, preclude a straightforward generalization. Also, many of these previous studies used still pictures as stimuli, leaving untested how individuals respond to dynamic social stimuli typical of natural environments. Critically, none of these studies have tested how experience-dependent factors can effect viewing patterns across individuals within nonhuman primate species. Thus, it remains unclear the extent to which within- and between-species variations overlap and how factors such as species and experience affect viewing patterns.

This study has two complementary goals. First, we examined individual and species differences in social orientating by presenting naturalistic movie stimuli to larger samples of human and nonhuman primates than previously tested (humans, bonobos, chimpanzees, orangutans, rhesus macaques). Experiment 1 aimed to extend the results from the previous studies and examined overall species similarities and differences in social orienting among rhesus macaques, three species of great apes (bonobos, chimpanzees, and orangutans), and humans. The movies depicted natural behaviors of conspecific and allospecific animals. Experiment 2 examined the effects of individual experience on within-species differences in social orienting. We tested three groups of chimpanzees housed at facilities that differed in their early experiences with media and cognitive experiments. We tested movies depicting natural behaviors of chimpanzees. We presented these same movies to three groups of humans differing in their expertise in observing chimpanzees (experts vs. novices) and in their experience with media in general (adults vs. preschoolers).

Second, we contrasted two methods for quantifying viewing patterns. One of the most common analytic strategies is quantifying the viewing times for predefined Areas-Of-Interests (AOIs). However, a clear shortcoming of this approach is that it may overlook attention to certain features that are salient to nonhuman participants but not to human researchers. An alternative, novel, data-driven approach consists of directly measuring gaze similarities using distances and correlations among individuals [5]. We contrasted these two approaches in this study. In the AOI viewing-time analysis, we first defined AOIs for the social features that previous studies typically included—faces, eyes, mouths, and action targets—to measure viewing times of these social features. We then used a Principal Component Analysis (PCA) to identify the components that best explained the observed variations across individuals and species. In the data-driven analysis, we estimated gaze similarities between each pair of participants, created a similarity matrix, and then performed Multi-Dimensional Scaling (MDS) to identify the dimensions that best explained the observed variations across individuals and species. Finally, we used a canonical correlation analysis to test the similarities between the components/dimensions derived from the two different analyses. If the major features distinguishing between individuals’ scanpaths were adequately captured by their viewing times for the defined AOIs, the two analyses would correlate with one another. Combining the two approaches allows us to characterize species’ similarities and differences thoroughly, and to confirm whether gaze toward AOI adequately describes the variations detected in the data-driven analysis.

## Experiment 1

We examined how bonobos, chimpanzees, orangutans, rhesus macaques, and humans view movies depicting various natural behaviors of these species and of nonprimate animals. Those behaviors included resting (with the individuals’ neutral faces), intense engagements among individuals such as playing and fighting (with the individuals’ emotional expressions), and extractive foraging such as manipulating foods and using tools. We predicted, in accord with previous studies [4–7, 15, 43–48], that species is the primary factor influencing individuals' unique viewing patterns of particular social features: faces, eyes, mouths, and action targets.

### Method

#### Participants

A total of 47 nonhuman primates (12 bonobos, 21 chimpanzees, 7 orangutans, and 7 rhesus macaques) and 12 humans participated in this study. An additional macaque was tested but not included in the analysis because of a calibration failure. All species lived in social groups. Twenty-eight apes (6 bonobos, 15 chimpanzees, 7 orangutans) lived in Wolfgang Köhler Primate Research Center (WKPRC) and 12 (6 bonobos, 6 chimpanzees) in Kumamoto Sanctuary (KS). Apes in these facilities had visual access to members of the other ape species. Macaques lived in a conspecific group at The Rockefeller University. All nonhuman participants had some experience watching movies (e.g., in the previous experiments or as enrichment), with KS chimpanzees being more experienced than the others (see Results and Experiment 2 for the effect of such experience). They were reared by their biological mothers or human caregivers in conspecific peer groups (see Table A in S1 File for further details about the participants). No ape or monkey participant showed a behavioral indication of vision deficit through our daily observation. Human participants were zoo workers at the WKPRC with extensive experience in interacting with nonhuman primates. All had normal or corrected-to-normal vision. No participant with neurological disorder or developmental delay was included. They were instructed to simply watch the movies as they normally would.

#### Ethics statements

Apes lived in Wolfgang Köhler Primate Research Center (WKPRC) and Kumamoto Sanctuary (KS). In both facilities, the living areas were large and complex enough for the apes to rest, exercise, and socialize with the group mates. The outdoor playground areas were larger than 200 m2 and were equipped with climbing trees, vegetation and enrichment devices. The indoor areas including sleeping rooms were larger than 100 m2. The apes received fresh fruits, vegetables, nuts and leaves distributed in three main meals and occasional enrichment programs. Water was available ad libitum throughout the day. They voluntarily participated in the study and were not food or water deprived. In KS, apes were tested in one of their sleeping or in a separate routine testing room (> 9 m2). In WKPRC, all apes were tested in one of their sleeping rooms (9 m2). No medical, toxicological or neurobiological research of any kind is conducted at KS or WKPRC.

Ape husbandry and research complied with the international standards in accordance with the recommendation of the Weatherall report “The use of non-human primates in research” and the institutional guidelines which are strictly adhered to the national laws of Japan or Germany [KS: Primate Research Institute “Guide for the Care and Use of Laboratory Primates 3rd Edition”, Wildlife Research Center “Guide for the Animal Research Ethics”] [WKPRC: “EAZA Minimum Standards for the Accommodation and Care of Animals in Zoos and Aquaria”, “WAZA Ethical Guidelines for the Conduct of Research on Animals by Zoos and Aquariums”, “Guidelines for the Treatment of Animals in Behavioral Research and Teaching” of the Association for the Study of Animal Behavior (ASAB)]. The study protocol was approved by the institutional committee of Wildlife Research Center (No. WRC-2014KS001A) and Max-Planck Institute for Evolutionary Anthropology.

All macaque procedures conformed to the NIH Guide for Care and Use of Laboratory Animals of the National Institutes of Health, and were conducted in accord with a local Institutional Animal Care and Use Committee (IACUC) protocol (#12585-H and #15849-H at The Rockefeller University). Monkeys were housed in a climate-controlled indoor colony in suites comprising 1–4 individuals. Monkey health was monitored daily, and monkeys were provisioned daily with biscuits, fresh fruits and vegetables, and behavioral enrichment including puzzle feeders.

Human adult participants were tested in a testing room located at the Max-Planck Institute for Evolutionary Anthropology (MPI-EVA), Leipzig, Germany. All agreed to and signed the written informed consent, which was in accordance of Helsinki Declaration and approved by the internal committee of MPI-EVA. For the preschooler participants, their parents were recruited by telephone from a database of parents who had volunteered to participate in developmental studies. All parents agreed the informed consent upon coming to the institute. They were tested in a testing room located at MPI-EVA. All agreed to and signed the written informed consent, which was in accordance of Helsinki Declaration and approved by the internal committee of MPI-EVA.

#### Apparatus

Apes at the two facilities, macaques, and humans watched the same movies in an eye-tracking system. The differences in eye-tracking setups were minimized as much as possible between different facilities. Eye movements of apes were recorded using an infrared eye tracker (60 Hz; down-sampled from X120/X300 eye-trackers; Tobii Technology AB, Stockholm, Sweden). This eye-tracker can record the participants’ eye movement without a head restraint device. WKPRC apes and KS bonobos were separated from the experimenter and the eye-tracker by a transparent acrylic panel (this panel does not add noises in the eye-movement recordings). To keep their heads relatively still, we let apes drink dripping grape juice from a nozzle attached to transparent acrylic panels. For the KS chimpanzees, one of the experimenters stayed inside the room, sat beside them, and lightly held their chins during the recording. Another experimenter stayed outside the room, with the eye tracker, and recorded the participants’ eyes through transparent acrylic panels. No explicit training was conducted for apes. Stimuli were presented using Tobii Studio software (version 3.2.1) at a viewing distance of 65–70 cm with a resolution of 1170×720 pixels (approx. 39×25 degree) on a 22-inch LCD monitor (1366×768 pixel). Human participants were tested in a standard office using the same setups of the eye-tracker and the monitor.

Eye movement of macaques was recorded using an infrared eye-tracker (60 Hz; ETL-200, ISCAN, MA, USA). They sat in a primate chair, with head position maintained via head prosthesis, and performed a gaze calibration routine. They were trained to fixate simple shapes for calibration in this and the other experiments. In addition, they were trained in the other experiments (but not in this experiment) to fixate at the center point of the monitor. Fluid rewards (water droplets) were delivered during calibration, and during movie viewing at 3-second intervals independent of the macaques’ visual behavior. Stimuli were presented using Presentation software at a viewing distance of 50 cm with a resolution of 1014x624 pixels on a 20-inch LCD monitor (1024x768 pixels; two macaques, Sam and Thor, were tested at a viewing distance of 57 cm with a resolution of 1202x754 pixels on the monitor, 1600x900 pixel; yet we confirmed that such differences did not affect the results; see below and Fig D in S1 File). These setups were adjusted so that the images occupied about the same visual angles as in the apes’ and humans’ settings.

We conducted calibration procedures previously established for apes, humans, and macaques at each facility [5, 51, 52]. For apes, automated calibration was conducted in Tobii Studio by presenting a small object or movie clip on two reference points. Although the number of these reference points was smaller than that used typically for human and monkey participants, we manually checked calibration accuracy after the calibration, by examining the discrepancies between the participant’s gaze and the 9 reference points presented on the screen, and repeated the calibration until those observed discrepancies became smaller than a degree. For human participants, automated calibration was conducted in Tobii Studio by presenting small objects at 5 reference points. Calibration was conducted for macaques in a Presentation software (Neurobehavioral Systems, California, USA) by presenting simple geometric shapes at 9 reference points. These procedures assured comparable accuracy of calibration for each species (typically within a degree), as detailed in the previous studies [5, 51, 52]. It should be noted that our control analysis ensured that the distribution of fixations around each defined Area-Of-Interest, which accommodate any calibration errors, was similar across the participant species (see Result and Fig B in S1 File).

#### Stimuli and procedure

Movies (total 9 minutes, 25 fps) depicted the natural behavior of conspecifics and allospecifics (obtained from ARKive.org). We prepared a total of 18 movies (each 30 seconds) featuring bonobos (3 clips), chimpanzees (3 clips), orangutans (3 clips), rhesus macaques (3 clips), and 3 nonprimate species (1 clip each of horses, dogs, and birds). Human movies were omitted in this study because the previous studies have confirmed similar eye movement patterns for human and allospecific images in adult human participants with experience interacting with nonhuman primates [5, 43, 44]. The contents of movies were selected so that they covered a wide range of species-typical behaviors of each primate. The three clips respectively depicted “actions”, “social interaction”, or “resting”. “Actions” included extractive foraging behavior such as manipulating objects (ground digging by bonobos and macaques, food washing by macaques), tool-using (stick-use by chimpanzees and orangutans, nut-cracking by chimpanzees), and eating the extracted foods. “Social interactions” included intense social engagement among individuals such as fighting (by bonobos, chimpanzees, and macaques) featuring threat and fearful facial and bodily expressions, copulating (by bonobos), and playing (by orangutans). "Resting" included calm, relaxed individuals, mainly showing the face. The non-primate movies depicted scenes of the species-typical behavior of those species (e.g., eating, fighting, flying, and galloping). Also, we presented participants with image-scrambled movies (3 clips) to obtain baseline data for the data-driven analysis (to control for the participants' default viewing biases to the screen). However, nonhuman participants only watched those movies for about half of the time that they spent watching the other movies, and human participants showed peculiar patterns of eye movement (i.e., kept looking at the center of the images). We thus did not use these data in the analysis. Instead, we used the time-shuffled eye movements (derived from the same trials presenting the same movies) as an alternative baseline for the data-driven analysis (see below). All movies were silent. Each ape viewed one movie (1 trial) per day (total 18 trials). If an ape became distracted during any given trial, that trial was dropped and the same trial was repeated on the next day (but no more than once). Each human and macaque viewed all movies consecutively in a single day, with a short blank period between movies. Yet, we did not particularly observe fatigue (or an increase in the percentage of off-screen gaze) in macaques (off-screen gaze was less than 30% in all trials) or in humans (less than 10% in all trials). The order of the movie presentation was counterbalanced across participants. See the video showing all stimulus movies and the superimposed gaze patterns in the first author's online repository (https://youtu.be/JLLW3ophuTc**).**

#### Data analysis

Viewing-time analysis. Areas-Of-Interest **(**AOIs) were defined for the eyes and mouth (when the scene focused on the face), head (when the scene focused on the whole body), and action targets as polygons around each feature of interest, frame-by-frame, by a primary coder using custom software. We did not define AOIs smaller than 1% or larger than 25% of the frame size (i.e. not defining the eyes/mouth when the whole bodies were zoomed-out, and not defining the body when the face was zoomed-in). The size of AOIs was approximately 20% larger than the objects of interest to accommodate small offsets which may derive from calibration errors or quick movements of objects in movies. Our control analysis ensured that small variations in the size of AOIs did not affect overall pattern of results (see Result and Fig B in S1 File). The "head" AOI was defined when the head appeared along with the whole body (and the eye/mouth AOIs were too small to be defined). The “action targets” included any goal targets of manual actions, including the foods being grabbed by hands (or pecked by a bird’s beak), the ground being dug, and the tools being manipulated. We then calculated viewing times for each category of AOI. We did not exclude off-screen gaze from this analysis (i.e. we used the raw, rather than proportion, viewing times) but excluded off-screen gaze in another, inter-individual distance, analysis and tested the similarities between these analyses (see below). Moreover, when we used such proportion data in this same analysis, we obtained the same pattern of results (Fig C in S1 File). See the video showing example frames and superimposed AOIs in the first author’s online repository (https://youtu.be/fb6N8-olJxk**).**

To reveal the components that best explained the observed individual and species variations, we performed Principal Component Analysis (PCA) on the viewing times for the eyes, mouth, head and action targets. To classify the participants by species, we performed a discriminant function analysis using the same data. We used permutation discriminant function analysis (pDFA) to control for the unequal number of participants in each species, using the code provided by R. Mundry [53]. This analysis samples an equal number of participants from each species (7 samples in this study, based on the minimal group size; 100 iterations) and classifies the sampled participants into predicted species based on the dependent variables. The remaining participants (i.e., non-sampled participants of large groups) were then used for external classification. The success rate of classification was compared with the chance level in a permutation test (i.e., the performance of the discriminant analysis on permuted data in which species identity had been randomly reassigned, 1000 iterations).

Inter-Individual Distance (IID) analysis. This data-driven analysis directly measures the gaze distances between the participants. Owing to its data-driven nature, this analysis benefits from some noise reduction. We did this in following way. First, we smoothed the series of horizontal and vertical gaze coordinates (60Hz) using a 100 ms (6 sample) moving average window to reduce the recording noises. We then calculated Inter-Individual Distances (IIDs), defined here as Euclidean distance between the gaze coordinates of a given pair, averaged across time-points for each movie clip. We did this for all pairs of participants. We excluded times of off-screen gaze from this analysis. To minimize a possibility that gaze similarities between participants derive from the similarities in default viewing biases (unrelated to the movie contents; e.g., central bias in humans [54]), we corrected the IIDs for baseline similarities. We calculated those baseline IIDs by shuffling the timestamps of a given scanpath 10 times and averaging over the repetitions. We then normalized the raw IIDs by dividing out the time-shuffled IIDs. These procedures created a similarity matrix. Based on this similarity matrix, we performed multi-dimensional scaling (MDS) and inferred the dimensions that best explained gaze similarities among participants. We took the first three dimensions for the analysis, based on the elbow of a scree plot [55]. Finally, we tested the similarities between the two analyses, using a canonical correlation analysis to compare these MDS dimensions with the PCA components derived from AOI viewing times.

### Results

**Fig 1:**
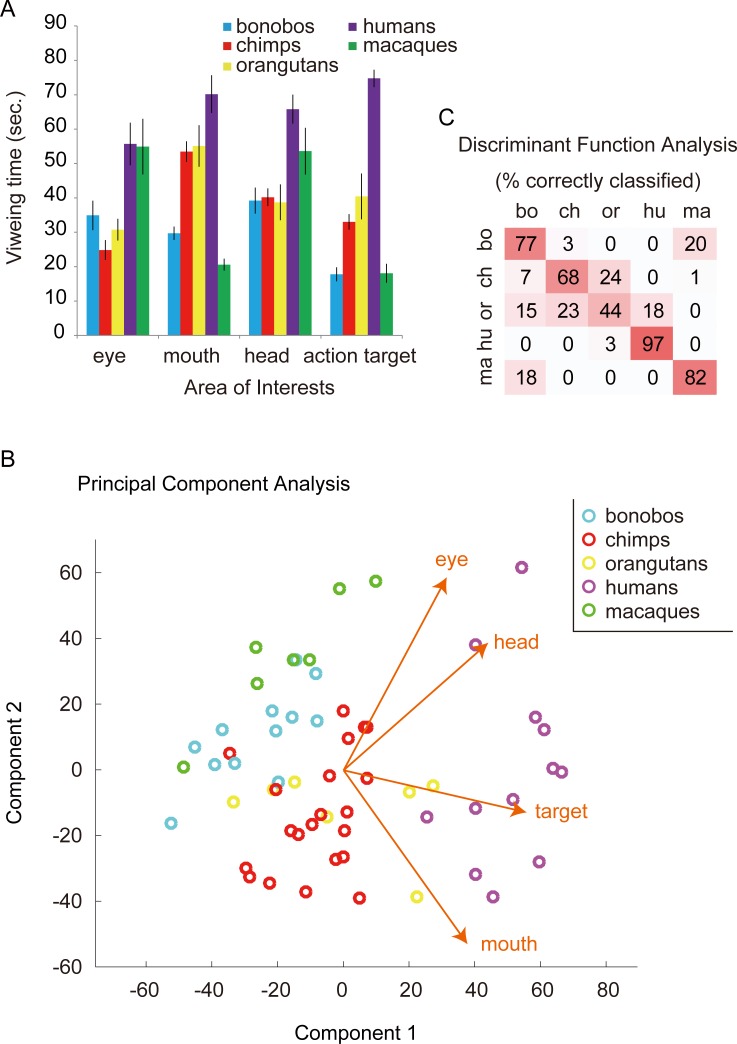
A. The total viewing times for Areas-Of-Interest (sec. ± SEM) summed across all movies in Experiment 1. Note that the “head” AOI was defined when the head appeared along with the whole body (and the eye/mouth AOIs were too small to be defined). B. Principal Component Analysis (PCA) map based on the viewing times for AOIs. The vectors in the map indicate the relative contribution of each viewing-time variable to the principal components (i.e., scaled PCA coefficients). C. The confusion matrix from permutation Discriminant Function Analysis (DFA) based on the viewing times for AOIs.

In the viewing-time analysis, we measured the participants’ viewing times for the Areas-of-Interest (AOIs) eyes, mouths, heads, and action targets. Fig 1A shows the viewing-time scores for each species summed across all movies. A MANOVA revealed highly distinct patterns across species in viewing times toward AOIs (F(16,156) = 11.1, p < 0.001, Wilk's Λ = 0.10) and also in viewing times to particular AOI categories (follow-up ANOVAs; eyes: F(4,54) = 9.40, p < 0.001, *η*^2^ = 0.41; mouth: F(4,54) = 22.21, p < 0.001, *η*^2^ = 0.62; head: F(4,54) = 9.03, p < 0.001, *η*^2^ = 0.40; action target: F(4,54) = 57.58, p < 0.001, *η*^2^ = 0.81; the alpha was set at 0.0125 with Bonferroni correction for number of comparisons).

We then used Principal Component Analysis (PCA) to identify the components that best explained the observed variation. We took the first two components in this analysis because they explained the majority (93.5%) of the observed individual differences (97.1% with the first three components). Fig 1B plots all the participants in these components. The largest coefficient of the first principal component corresponded to the viewing time for action targets (0.71), followed by head (0.44) and mouth (0.42). The largest coefficient of the second component corresponded to the viewing times for eyes (0.67) and mouth (-0.63; see the vectors in Fig 1B for these coefficients).

**Fig 2:**
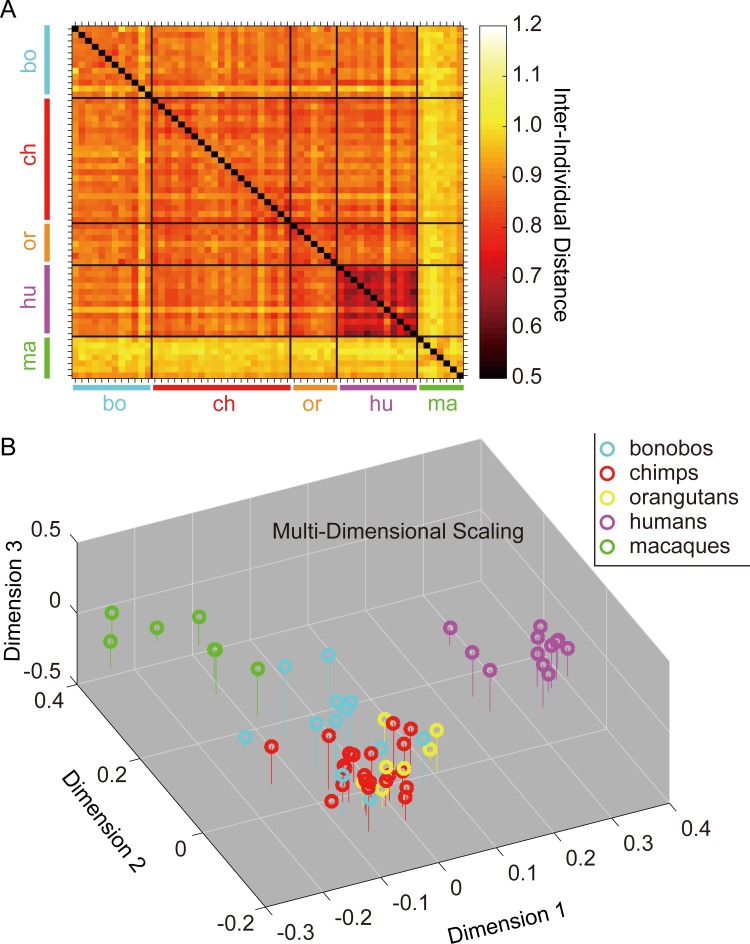
A. The distance matrix showing the Inter-Individual gaze Distances (IIDs, corrected for the chance-level similarities) averaged over all movies in Experiment 1. Lower values indicate better similarities among participants. B. Multi-Dimensional Scaling (MDS) map based on this distance matrix.

In the Inter-Individual Distance (IID) analysis, we measured IIDs between all pairs of participants and then created the gaze-similarity matrices for all movies. Fig 2A shows the gaze-similarity matrix averaged for all viewed movies (see Fig A in S1 File for the data for each viewed species’ movies). We then used Multi-Dimensional Scaling (MDS) to identify the dimensions that best explain the observed variation. Fig 2B shows the distribution of all participants in the 3D space based on this MDS analysis.

We then tested the similarity between data derived from the PCA analysis (first two components) and from the MDS analysis (first 3 dimensions) using a canonical correlation analysis. We found that the canonical correlation was 0.81 and 0.61 for the first and second canonical dimensions, respectively. Both canonical dimensions were significant (1^st^ to 2^nd^: F(6,108) = 20.6, p < 0.001, Wilk's Λ = 0.22; 2^nd^: F(2,55) = 16.7, p < 0.001, Wilk's Λ = 0.62). The first canonical dimension was most strongly influenced by the first MDS dimension (standardized canonical coefficient 0.99) and the first PCA component (0.998). The second canonical dimension was most strongly influenced by the second and third MDS dimensions (0.73, 0.68) and the second PCA component (0.999). Note that IID analysis excluded off-screen fixations from the analysis (i.e., used on-screen gaze distances), while the viewing-time analysis did not (i.e., used raw, not proportion, viewing times). Thus, the observed similarity between the two results ensured that the species differences in overall levels of attention to the movies (i.e., on-screen viewing times; bonobos were slightly less attentive than the other species; see Table B in S1 File) cannot alone explain those in the viewing patterns of specific social features (also see Fig C in S1 File for the replication of the same results with a proportion measure excluding off-screen gaze). Moreover, it indicates that the major features distinguishing between individuals’ scanpaths were adequately captured by their viewing times for the defined AOIs.

**Fig 3:**
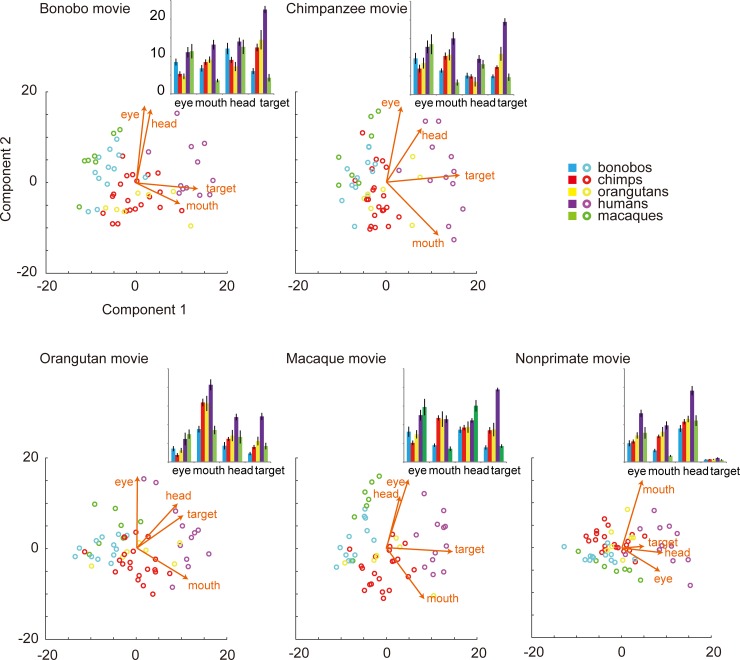
The total viewing times for AOIs (sec. ± SEM) for each species' movies and the PCA map based on the viewing times for AOIs in Experiment 1. The vectors in the map indicate the relative contribution of each viewing-time variable to the principal components (i.e., scaled PCA coefficients).

To test the clustering of participants based on their species, we performed a permutation Discriminant Function Analysis (pDFA; [53]) using the viewing times for AOIs (the data for all movies were averaged). The classification based on the participants’ species was highly successful (83.4%; chance-level, 37.9%; p < 0.001). Most misclassifications occurred between orangutans and chimpanzees/humans and between bonobos and macaques (Fig 1C). Fig D in S1 File presents the names of all participants in the PCA graph and Table A in S1 File details the properties of each participant. The inspection of the remaining misclassified participants did not reveal common properties (including living facility, sex, age class, or whether mother- or human-reared; yet WKPRC and KS chimpanzees somewhat differed from one another in their viewing patterns of the action targets; this group difference was further examined in Experiment 2). Classification based on the participants’ species was successful with the data for any given depicted species’ movie (Table 1). This indicates that each species viewed social features (eyes, mouth, face, and action targets) of each depicted species similarly across the movies (Fig 3) (although bonobos viewed the conspecific movies somewhat for a longer time than the allospecific movies; see Table B in S1 File).

**Table 1:** Classification accuracy from permutation Discriminant Function Analysis based on the viewing times for AOIs.

	Original %	External %
Overall	73.7 (41.4)***	58.8 (20.4)***
Bonobo movie	73.6 (41.5)***	47.8 (19.9)**
Chimpanzee movie	75.8 (41.9)***	59.2 (20.1)***
Orangutan movie	69.1 (41.5)***	52.1 (20.1)**
Macaque movie	77.0 (41.6)***	56.6 (19.8)**
Nonprimate movie	70.9 (41.0)**	52.2 (20.5)***

Chance-level classifications are shown in parentheses (***p < 0.001, **, p < 0.01 in permutation test).

Finally, there may be a concern that potential differences in calibration error between species (due to the procedural differences between facilities) may affect the pattern of species differences to some degree. Yet, this was not an issue here. First, we generated matching results through two different analyses with different sensitivity to calibration noise: the AOI viewing-time and inter-individual distance analyses. Moreover, we conducted a control viewing-time analysis (Fig B in S1 File) manipulating the size of AOIs (shrinking or expanding the size up to 20%) and confirmed that such manipulation did not change the pattern of species differences in the viewing-time data. This result indicates that the distribution of fixations (including any calibration errors) around each defined AOI was similar across the participant species.

### Discussion

Overall, bonobos, chimpanzees, orangutans, rhesus macaques, and humans exhibited similar yet highly discriminable gaze patterns to the movies. We found a strong correlation between the results from the data-driven IID analysis and those from the AOI viewing-time analysis. The use of two different analytical approaches revealed that the viewing patterns for the models’ face, eyes, mouth, and action targets satisfactorily characterized overall gaze similarities. It also revealed that variations in overall levels of attention to the movies (somewhat lower in bonobos than in the other species) cannot explain variations in viewing patterns across social features, because one of our analyses excluded the off-screen fixations from the analysis, while the other did not.

More specifically, we found that humans viewed the action targets for a much longer time than apes and macaques. Bonobos viewed the eyes for a longer time (and the mouth for a shorter time) than chimpanzees and orangutans. Chimpanzees and orangutans viewed the mouth and the action targets for a longer time than bonobos. Macaques’ viewing patterns were somewhat similar to bonobos in the sense that they viewed the eyes for a longer time than chimpanzees and orangutans, although the data revealed clear differences between bonobos and macaques; with the latter viewing the eyes even longer, and the mouth even shorter, than the former. These results are largely consistent with the previous studies [4, 43, 44], although some results are unexpected (e.g. monkey-ape difference). We will discuss the implications of these results in General Discussion.

Consistent with the previous eye-tracking studies [4, 43, 44], we found that the observed species-typical viewing patterns were relatively independent of whether the presented species was conspecifics or allospecifics. This result suggests that such species-typical patterns likely reflect their general responses to the social features that are commonly present in animate agents (e.g., face-like shapes, contingent motions). It is noteworthy that bonobos viewed the conspecific (and also the chimpanzee) movies for a longer time than the allospecific movies (Table B in S1 File). This result suggests that bonobos might have a higher interest in conspecific than allospecific movies, although their viewing bias for each social feature (e.g., eyes versus mouth) was highly similar for both types of movies.

## Experiment 2

Experiment 1 revealed some differences in the viewing patterns of action targets of two groups of chimpanzees (WKPRC vs. KS1). Several studies have documented that early experiences with the social and physical environment are especially influential in the adulthood behaviors of great apes. Enculturated apes reared by humans in human cultural environment performed particularly well at tasks requiring joint attention with human experimenters [33–35]. Additionally, deprivation of social and physical experience in early life adversely affects social behaviors in adult chimpanzees [56–59]. In Experiment 2 we further examined the role of experience on viewing patterns by presenting movies of chimpanzee natural behavior to three groups of chimpanzees differing in their early social and physical experiences (individuals were reared in three different facilities). One group of chimpanzees (WKPRC) had standard experiences with media and cognitive experiments, another group (KS1) had more extensive early experiences with media, cognitive experiments, and tool-using training, and the third group (KS2) had relatively little early experience with media and cognitive experiments, and relatively little social and physical enrichment during their development (prior to arriving at the sanctuary). In line with the findings from Experiment 1, we expected that WKPRC and KS1 groups differ from one another in their viewing patterns for the action targets in the movies. Additionally, we expected that the KS2 group differ from the other two groups in their general viewing patterns for the social features in the movies.

We also presented the same movies to three groups of humans differing in their expertise in observing chimpanzees or in their experiences with media in general; expert fieldworkers who had an extensive experience of observing chimpanzees in the wild, novice researchers who did not have an experience of working with chimpanzees, and preschooler (novice) children who likely had fewer experiences of watching movies in general. We expected to observe the effect of expertise between the first two groups and a more general effect of media exposure between the first two and the last groups.

### Method

#### Participants

A total of 26 chimpanzees and 58 humans participated in this study. An additional human was tested but not included in the analysis because of a recording failure. Chimpanzee participants consisted of three groups differing in their early experiences (“early” defined here as the infancy and the juvenile period, roughly covering the first nine years). Fourteen WKPRC chimpanzees had moderate experience participating in cognitive experiments and some experience watching movies in previous eye-tracking experiments. They were either reared by their biological mothers or human caregivers (and conspecific peers; See Table C in S1 File for further details). Six KS chimpanzees (KS1 group) were recently moved to KS from the Great Ape Research Institute, Okayama, Japan. They had extensive experience participating in various cognitive experiments and watching movies in experiments and as enrichment. They were also trained, since youth, to perform complex tool-use behaviors, including nut-cracking behaviors (while WKPRC chimpanzees were not). WKPRC and KS1 chimpanzees were either reared by their biological mothers or human caregivers and conspecific peers (see Table C in S1 File for further details). The other six KS chimpanzees (KS2 group) had almost no experience participating in cognitive experiments or watching movies. They had been housed in isolation for biomedical research and reared by human caregivers during the infancy and juvenile periods. They arrived at Kumamoto Sanctuary between 1980 and 2000 to be integrated into a more naturalistic conspecific social group. Note that, after the adoption to the sanctuary, KS2 chimpanzees live in a socially- and physically-enriched environment as do the other participant chimpanzees (see SI for the details about enrichments and the ethical statements). No ape participant showed a behavioral indication of vision deficit through our daily observation.

Human participants consisted of three groups differing in their experience watching chimpanzee behavior and movies. Eighteen humans were professional field-worker researchers who had expertise working with chimpanzees in their wild habitats. Twenty humans were researchers who had no experience working with chimpanzees. Thirteen expert humans (of 18) and 10 novice humans (of 20) reported that they have already seen the movie used for our stimuli, and thus knew the basic stories used in the movies, yet we confirmed that this factor did not affect the results (see below). Most had European or North-American origins (4 expert humans were from Japan; yet, they were not different from other experts, as shown below). They were instructed simply to watch the movies as they normally would. Twenty humans were preschoolers aged between 5 and 6 years (mean age 5.6 ± 0.29). Their parents reported that no preschooler participant watched the “chimpanzee” movie but had some experiences of watching movies of nonhuman animals in general, and that all had regulated opportunities of watching TV and cinemas made for juveniles/adults (see SI for the ethical statements and Table C in S1 File for further details about participants). All had normal or corrected-to-normal vision. No participant with neurological disorder or developmental delay was included.

#### Apparatus

WKRPC and KS1 chimpanzees and humans were tested with the same eye-tracking setup as those used in Experiment 1. KS2 chimpanzees were tested with the same eye-tracking setup as those used for WKPRC apes and KS bonobos in Experiment 1 (i.e. with transparent panels between the participant and the eye-tracker/the experimenter).

#### Stimuli and procedure

Movies (total 6 minutes, 25 fps) depicted the natural behavior of chimpanzees in the wild (taken from *Chimpanzee* by Disney Nature). We prepared a total of 12 movies (each 30 seconds) featuring resting, grooming, eating, tool-using, playing and fighting (2 clips for each). Resting clips depicted calm, relaxed individuals, mostly faces. Grooming and play clips depicted grooming and playing bouts between dyads. Fighting clips depicted agonistic episodes among individuals that included threat and fear facial expressions. Eating clips depicted individuals grabbing and consuming food. Tool-using clips depicted individuals using a probe-stick to extract the insects inside the wood and a hammer (a log) to crack open nuts on an anvil. No sound accompanied the movie images. Each ape viewed one movie (1 trial) per day (total 12 trials). If an ape became distracted during any given trial, that trial was dropped; and the same trial was repeated on the next day (but no more than once). Each human viewed all movies consecutively in a single day, with a short blank period between movies. Yet, we did not particularly observe fatigue (or a strong increase in the percentage of off-screen gaze) in human adults or preschoolers (off-screen gaze was less than 10% in all trials). The order of the movie presentation was randomized for each participant. See the video showing all stimulus movies and the superimposed gaze patterns in the first author's online repository (https://youtu.be/KfVqWAP-D6Q**).**

#### Data analysis

We used the same method as Experiment 1 for the data analysis except that we distinguished between the “in-hand action targets” and “distal action targets” for the definition of AOIs in this study, because the movies included a long sequence of nut-cracking behaviors. The “in-hand action targets” covered any goal targets of manual actions including the foods being grabbed by hands, the ground being dug, the body part being groomed, and the tools being manipulated. The “distal action targets” are the nuts being placed on anvils and cracked open by chimpanzees with hammers.

### Results

**Fig 4:**
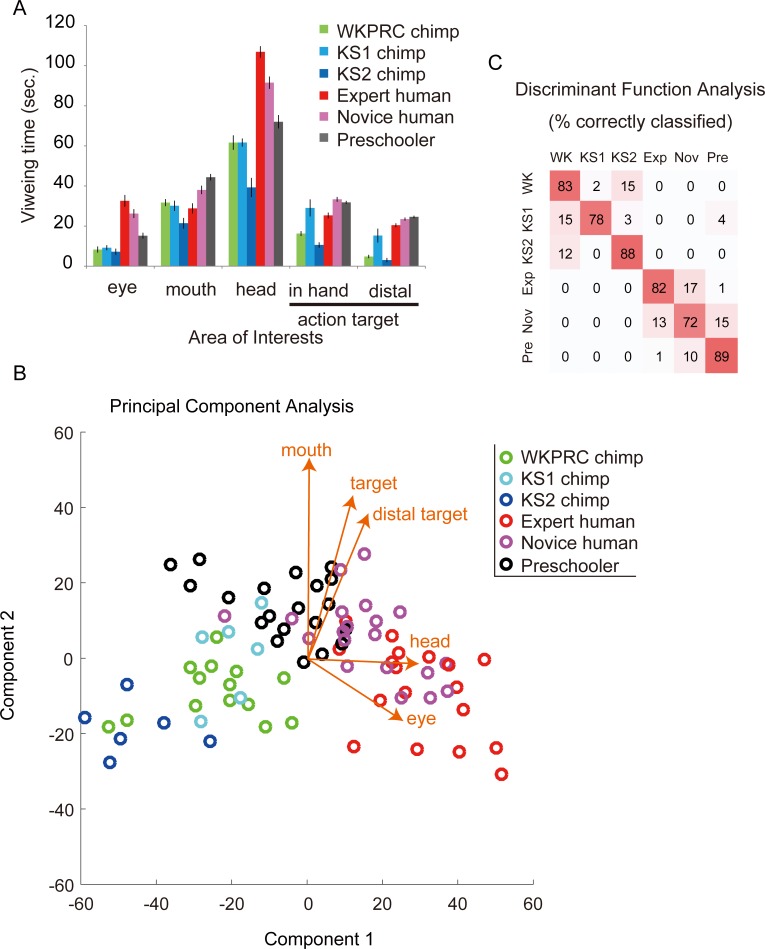
A. The total viewing times for AOIs (sec. ± SEM) summed across all movies in Experiment 2. B. Principal Component Analysis (PCA) map based on the viewing time for AOIs. The vectors in the map indicate the relative contribution of each viewing-time variable to the principal components (i.e., scaled PCA coefficients). C. The confusion matrix from permutation Discriminant Function Analysis (DFA) based on the viewing times for AOIs.

In the viewing-time analysis, we measured the viewing times for AOIs comprising eyes, mouths, heads, in-hand action targets, and distal action targets (Fig 4A). A MANOVA revealed highly distinct patterns across groups (F(25,276) = 17.1, p < 0.001, Wilk's Λ = 0.03) and also in viewing times to particular AOI categories (follow-up ANOVAs; eyes: F(5,78) = 20.51, p < 0.001, *η*^2^ = 0.57; mouth: F(5,78) = 11.19, p < 0.001, *η*^2^ = 0.42; head: F(5,78) = 39.37, p < 0.001, *η*^2^ = 0.72; action target: F(5,78) = 31.78, p < 0.001, *η*^2^ = 0.67; distant action target: F(5,78) = 71.63, p < 0.001, *η*^2^ = 0.82; the alpha was set at 0.01 with Bonferroni correction for a number of comparisons). We then used PCA to identify the components that best explained the observed variation. We selected the first two components in this analysis because they explained the majority (89.8%) of the variation (97.0% with the first three components). Fig 4B plots all the participants as a function of these two components. The largest coefficient of the first principal component corresponded to the viewing time for the head (0.88) followed by the eyes (0.41). The largest coefficient of the second component corresponded to the viewing time for the mouth (0.71) followed by the in-hand action target (0.49), and the distal action target (0.42; see the vectors in Fig 4B for these coefficients).

Next, as in Experiment 1, we measured IIDs between all pairs of participants, created the gaze-similarity matrix, and identified the three dimensions (based on an elbow of the scree plot) that explain the observed individual variations using MDS (see Fig E in S1 File for the plot). We then tested the similarity between the data from the AOI-PCA analysis and those from the IID-MDS analysis using a canonical correlation analysis based on the first three dimensions of MDS and the first two (i.e., most influential) components of the PCA. We found that the canonical correlation was 0.91 and 0.78 for the first and second canonical dimensions, respectively. All these canonical dimensions were significant (1^st^ to 2^nd^: F(6,158) = 75.5, p < 0.001, Wilk's Λ = 0.07; 2^nd^: F(2,80) = 63.4, p < 0.001, Wilk's Λ = 0.39). The first canonical dimension was most strongly influenced by the first MDS dimension (standardized canonical coefficient -0.92) and the first PCA component (0.999). The second canonical dimension was most strongly influenced by the second MDS dimensions (-0.96) and the second PCA component (0.999).

Finally, we performed a permutation Discriminant Function Analysis (pDFA, [53]) using the viewing times for Area-Of-Interests. The classification based on the participants’ group was highly successful (81.9%; chance-level, 43.1%; p < 0.001). The majority of misclassifications occurred between novice and expert humans, between novice adults and preschoolers, and between WKPRC chimpanzees and KS1 or KS2 chimpanzees (Fig 4C). Misclassifications across species were rarely observed, although some occurred between KS1 chimpanzees and preschoolers. Fig F in S1 File presents the names of all participants in the PCA graph and Table C in S1 File details the properties of each participant. The inspection of the remaining misclassified participants did not reveal common properties, including sex, age class, whether the chimpanzee was reared by their biological mother or human caregivers/conspecific peers, and whether the human participant had previously seen the movie. Note that all participants were from Western countries except some Japanese experts, HE15-19, who did not differ from the other experts (Fig F in S1 File). These results thus indicate that experimentally-selected group (or species) was the major factor in this classification.

### Discussion

Overall, several groups of chimpanzees and humans exhibited similar yet highly discriminable gaze patterns during viewing of social movies. Consistent with Experiment 1, we found a strong correlation between the data from the data-driven IID analysis and the AOI viewing-time analysis. As in Experiment 1, the use of two different analytical approaches revealed that variations in attention to the models’ face, eyes, mouths, and action targets could satisfactorily characterize overall gaze similarities. Also consistent with Experiment 1, we confirmed that species was the primary factor affecting the observed variations, although there were substantial differences within each species, notably relating to the participants’ rearing and experimental histories.

More specifically, KS1 chimpanzees viewed the models' action targets (both in-hand and distal) longer than the other chimpanzee groups. Moreover, KS2 chimpanzees viewed all social features for a shorter time than the other chimpanzees (i.e., they viewed nonsocial features proportionally for a longer time than did the other chimpanzees). Expert humans viewed the faces and eyes of model chimpanzees longer (and the mouth and action targets for a shorter time) than the other humans. Moreover, children viewed the models’ action targets longer (and the models’ faces and eyes for a shorter time) than adults.

## General discussion

We examined individual and species variation in the viewing patterns of movies depicting the natural behaviors of nonhuman primates in rhesus macaques, three species of great apes (bonobos, chimpanzees, and orangutans), and humans. We found that social orienting was both individually-variable and species-typical across human and nonhuman primates. Also, we found that variation in the viewing of the models’ faces, eyes, mouths, and action targets can distinguish both the species and experiences of the viewer. This result supports the idea that attention to others’ eyes and their manual actions are related to key aspects of social cognition in human and nonhuman primates [1, 60].

### Gaze toward action targets

Why did individuals and species vary in their viewing patterns in the observed ways? Multiple factors likely contribute to shaping such variation. Let us start by discussing observed differences in viewing targets of depicted actions. In this study, human participants viewed the action targets of any model animal for a much longer time than did the other primate species in both Experiments 1 and 2. In general, humans should be regarded as a special class of participants among the tested primates, because our stimulus movies were created in the human cultural environment, e.g., under specific conventions of cinematography [61]. One interpretation is thus that human participants, presumably even preschoolers, were much more accustomed than nonhumans to watching movies, and therefore better understood (and hence more actively viewed) the goals of depicted actions. The action targets in our stimuli were typical goal targets of manual actions by primate models, including foods being grabbed by hands, tools being manipulated, and nuts being placed on an anvil for cracking by a chimpanzee’s hammer. Moreover, some of the movie scenes contained complex configurations (e.g., zoomed-in manual movements). Humans should understand such movie content readily due to their unique experiences with cinematography, or should at least expect movies to provide some interesting and conceptually-related information across scenes.

Importantly, in Experiment 2, those chimpanzees with extensive early experiences with media (KS1) also viewed the models' actions for a longer time than the other chimpanzee groups. As in humans, their experiences with visual media may have enhanced their understandings and expectations about movie contents. Also, their early experience with cognitive experiments and training in tool-use, including nut-cracking, could have enhanced their understandings of movies and their attention to the distal goal targets (i.e., nuts). These results may be related to previous reports that "enculturated" chimpanzees are particularly attentive to human experimenters' action targets [33–35]. On the contrary, those chimpanzees who experienced relatively impoverished social and physical environment during their youth (KS2) viewed the depicted actions (and eyes) for a shorter time than other chimpanzee groups. Therefore, one candidate factor affecting the observed variation in the viewing of action targets may be related to our participants’ unique experiences with the human environment, including media viewing, tool use and cognitive testing.

Then, why did nonhuman species (with similar experiences) differ from one another? In Experiment 1, we observed that chimpanzees and orangutans viewed the models’ targets of manual actions for a longer time than did bonobos and macaques. One possibility is that bonobos and macaques were much more attentive to the models’ faces and eyes than actions, and thus could not spend much time in viewing the other features because of a time trade-off. However, this possibility is unlikely because (unlike humans) their on-screen viewing times to the movies did not reach to the ceiling level; this means that bonobos and macaques viewed elsewhere (including backgrounds and off-screen) instead of viewing the models’ manual actions.

Our results may be related to the previous observation that bonobos and rhesus macaques, unlike chimpanzees, orangutans, and humans, do not use tools in foraging contexts or show clear evidence of cultural transmission of tool-using in the wild [62, 63]. Thus, another possibility is that, similarly to what we discussed above, bonobos and macaques may have more poorly understood the models’ manual actions depicted in the movies and hence attended them less actively than did chimpanzees and orangutans. However, at least for bonobos, this explanation is inconsistent with the previous evidence. Studies have shown that bonobos can perform tool-using behaviors as dexterously as the other primate species if they have an opportunity to do so in a laboratory [64–66]. Also, researchers largely agree that motivational factors rather than competence better explain the absence of tool-using behaviors in bonobos living in the wild [62, 67]. Moreover, studies have shown that bonobos follow a model’s gaze more sensitively than the other ape species and consequently attend more to the target objects in such situations [10, 68]. Studies also have shown that bonobos are comparable to the other ape species in their performances of anticipatory looking to the agent’s manual reaching [14, 69]. Therefore, a more plausible explanation for our results with bonobos is that they were simply less interested than chimpanzees and orangutans in others’ manual actions due to their unique motivation and temperament [64]. It is also possible that their unique experiences during development, such as more limited opportunities for observing conspecifics’ manual actions than the other ape species, may have further discouraged them from gazing toward complex actions. However, it should be noted that this same explanation may not to apply to macaques. It is certainly likely that macaques understood the models' manual actions less well than did apes. Even so, it is unlikely they failed to understand simple actions such as macaques handling food, especially given previous studies showing that macaques can learn from conspecifics’ actions in natural experiments [70] and that their mirror-neuron system responds to both their own actions and actions performed by others [71]. Overall, motivational factors rather than competences likely explain the observed variations in the viewing of the models’ action targets across species.

### Gaze toward faces

Next, why did individuals and species differ in their viewing patterns of the model’s eyes and mouth? Regarding the (within-species) individual differences, in Experiment 2, we observed that attention to the eyes and mouth varied to a larger extent among humans than chimpanzees. Specifically, expert field-workers of chimpanzees viewed the face and eyes of model chimpanzees for a longer time than did novice researchers and preschoolers (and the mouth and action targets for a shorter time presumably due to a time trade-off). One interpretation of this result is that experts habitually attend to chimpanzees' faces and eyes to individuate chimpanzee faces. Specialization for processing and individuating particular faces or exemplars of inanimate objects (e.g., cars) is one of the well-known effects of expertise [72]. Our expert participants may be trained to individuate chimpanzee faces, or at least be more motivated than novices to individuate chimpanzees faces, and therefore may have attended to their faces more strongly than novices in the movies. In contrast, our preschooler participants’ inexperience with allospecific movies, or movies in general, may have discouraged them from attempting to identify individuals. Their inexperience may have instead motivated them to watch unfamiliar models’ performing certain actions. The observed adult-child differences in humans may be also related to certain developmental changes in social attention, in that adults may have a stronger tendency of looking at face and eyes of both conspecifics and allospecifics. This aspect cannot be fully examined in nonhuman primates in our study because most of our nonhuman participants were adults; the few juvenile participants did not obviously differ from the adult participants (Figs D and F and Table A and C in S1 File).

In Experiment 2, we also observed that the chimpanzees who underwent relatively impoverished social and physical environment during their youth (KS2) showed a decreased level of attention to all social features including face and eyes (i.e., they viewed nonsocial features proportionally for a longer time than the other groups). This pattern could derive from their lack of experience in watching movies or participating in cognitive experiments more generally. Given that early social deprivation adversely impacts social behaviors in chimpanzee adults [56–59], it is also likely that their reduced experience in communicating with conspecifics (and social agents in general) during their early lives discouraged them from attending to chimpanzees in the movies. Overall, therefore, individuals’ unique experiences likely affected their patterns of gaze toward eyes and mouths.

Why, then, did nonhuman species with similar early experiences differ from one another in the viewing of eyes and mouth? We observed that in Experiment 1, bonobos and macaques viewed the eyes for a longer time than the mouth, while chimpanzees and orangutans showed an opposite pattern. Given that, in a previous study, orangutans (the same participants as in this study) showed a similar viewing pattern for the face and eyes as gorillas [44], it is likely that bonobos are exceptional among great apes in their viewing patterns of the face and the eyes. Importantly, in Experiment 2, while the time spent viewing the eyes and the mouth varied to a large extent within the human species, it varied only to a small degree among the chimpanzee participants. Therefore, at least in chimpanzees, the observed viewing bias should reflect some inherent species-typical characteristic. Several previous studies may help to identify the nature of this trait. First, in humans, increased motivation to affiliate with particular others can lead to an increased level of eye contact with them [73]. Bonobos live in a more egalitarian society and exhibit more frequent and diverse affiliative behaviors towards social partners than do chimpanzees [74]. Thus, their general affiliative attitudes toward others may have led them to attend to others’ eyes than chimpanzees (and orangutans). Second, previous studies reported that bonobos and chimpanzees differ in brain areas implicated in social interaction [75], which were activated, in humans, when engaging eye contact [17]. Third, previous studies reported bonobos and chimpanzees differ in their endocrine systems. Bonobos have a lower level of prenatal androgens than do chimpanzees [76, 77], which is known to cause an increased level of eye contact in humans [28]. Bonobos and chimpanzees are also known to differ in their oxytocin- and vasopressin- receptor genes [78]; in humans and macaques, a higher level of oxytocin is reported to cause an increased level of eye contact [29, 30]. Therefore, bonobos may differ from chimpanzees (and possibly also from orangutans) in their psychobiological characteristics affecting the pursuit and tolerance of eye contact with others.

Interestingly, macaques viewed the models’ face and eyes for a longer time and the mouth for a shorter time than any other species, including bonobos. Our macaques viewed the models’ mouth and action targets very little; thus, overall, they almost exclusively viewed the models’ eyes among all social features in the presented movies. Such strong viewing bias to eyes (versus mouth) is consistent with previous studies [5–7, 45–48]. However, it was somewhat surprising that they did so even more than great apes in this study, because some researchers believe that prolonged eye contact is more commonly observed in great apes than in rhesus macaques [79]. There are several possibilities that could explain this result. First, our macaques, unlike our apes, had previously received fixation training. Thus, one possibility is that such different prior training may have encouraged them to search for certain salient stimuli (e.g., faces or eyes) as cues that could produce rewards. However, note that we did not reward the macaques for their viewing of any particular social features in this study. Also, the eye viewing patterns exhibited by the macaques in the previous studies with different training histories (or no reported prior training) were very similar to those exhibited by our macaques in this study [5–7, 45–48]. Therefore, overall, it is unlikely that their viewing patterns derive solely from their training histories.

The second possibility is that, like bonobos, a high level of social tolerance led them to focus on the models’ eyes more than the other species. However, this is unlikely because rhesus macaques live in a relatively despotic society [80] and make eye contact with conspecific adults in affiliative contexts less frequently than other macaque species ([81, 82] but see [83]). The third possibility is that, rather than tolerance, vigilance led our macaques attend to the models’ eyes more than the other species. In general, attention to eyes is enhanced in both affiliative and threating situations [1, 73]. Although it is reported that tolerance enhances attention to the eyes of others in macaques [29, 83], it is also reported that vigilance enhances their attention to the eyes (or the attentional status) of others such as when an experimenter maintains eye contact with them at a close distance [81]. Therefore, our macaques may have been more vigilant than apes to our movie stimuli and hence monitored the eyes of potentially threatening models exclusively in the movies. Finally, it is likely that the differences in rearing experience and the level of understanding of movie contents complicate a direct comparison between monkeys and great apes. Future studies should address this question by testing multiple species of monkeys using eye-tracking. It would be especially interesting to examine how the tolerance levels of social systems in closely-related macaque species (despotic versus egalitarian societies [80]) affect their distinct viewing of the eyes and the mouth.

### Conclusion

Lastly, from an animal welfare perspective, it is important to highlight that the patterns exhibited by chimpanzees who had poor experiences with media, cognitive experiments, and social and physical enrichments in youth. These chimpanzees had been isolated from their mothers and conspecifics and reared by human caregivers at a biomedical laboratory during their infant and juvenile periods, and only later they were transferred to more naturalistic groups in sanctuaries. Previous studies found that impoverished early social experiences negatively affect social behaviors of chimpanzees in general [56–59], but importantly, not all chimpanzees reared were affected in similar ways [59]. Thus, one possibility raised by our results is that the tests with eye movements can be used as a diagnostic tool to assess psychological differences across chimpanzee individuals to offer individualized care for those animals; for example, when they are integrated into more naturalistic social groups.

In summary, we found that although great apes, humans, and macaques view social movies overall similarly, individuals and species also have unique viewing patterns for several key social features (i.e., eyes, mouths and action targets). Also, we found that individual experiences and species-typical motivation and temperament explain some of the observed individual and species differences. This suggests that the underlying mechanisms affecting variation in social attention are similar across species. From an evolutionary perspective, our results suggest that closely-related primate species can acquire particular attentional biases relatively rapidly through ontogeny and evolution based on shared mechanisms. Such attentional biases might help them to learn effectively from the social environment and enhance their chances of survival and reproductive success.

## Supporting information

S1 File Supporting Tables, Figures, and Movie links. (DOCX)

## References

[1] Emery NJ. The eyes have it: The neuroethology, function and evolution of social gaze. Neurosci Biobehav Rev. 2000;24(6):581–604. doi: 10.1016/S0149-7634(00)00025-7 10940436 10.1016/s0149-7634(00)00025-7

[2] Klein JT, Shepherd SV, Platt ML. Social attention and the brain. Curr Biol. 2009;19(20):958–62. doi: 10.1016/j.cub.2009.08.010 19889376 10.1016/j.cub.2009.08.010 PMC3387539

[3] Kano F, Call J. Great ape social attention In: Shigeru W, M H, T S, editors. Evolution of Brain, Cognition, and Emotion in Vertebrates. Brain Science. Tokyo: Springer; 2017 p. 187–206.

[4] Kano F, Hirata S, Call J. Social attention in Pan: bonobos exhibit more eye contacts than chimpanzees. PLoS One. 2015;10(6):e0129684 doi: 10.1371/journal.pone.0129684 26075710 10.1371/journal.pone.0129684 PMC4468221

[5] Shepherd SV, Steckenfinger SA, Hasson U, Ghazanfar AA. Human-monkey gaze correlations reveal convergent and divergent patterns of movie viewing. Curr Biol. 2010;20(7):649–56. doi: 10.1016/j.cub.2010.02.032 20303267 10.1016/j.cub.2010.02.032 PMC2855404

[6] Mosher CP, Zimmerman PE, Gothard KM. Neurons in the monkey amygdala detect eye contact during naturalistic social interactions. Curr Biol. 2014;24(20):2459–64. doi: 10.1016/j.cub.2014.08.063 25283782 10.1016/j.cub.2014.08.063 PMC4253056

[7] Guo K, Robertson RG, Mahmoodi S, Tadmor Y, Young MP. How do monkeys view faces? A study of eye movements. Exp Brain Res. 2003;150(3):363–74. doi: 10.1007/s00221-003-1429-1 12707744 10.1007/s00221-003-1429-1

[8] Tomasello M, Call J, Hare B. Five primate species follow the visual gaze of conspecifics. Anim Behav. 1998;55(4):1063–9. doi: 10.1006/anbe.1997.0636 9632490 10.1006/anbe.1997.0636

[9] Shepherd SV. Following gaze: gaze-following behavior as a window into social cognition. Front Integ Neurosci. 2010;4:e5 doi: 10.3389/fnint.2010.00005 20428494 10.3389/fnint.2010.00005 PMC2859805

[10] Kano F, Call J. Cross-species variation of gaze following and conspecific preference among great apes, human infants and adults. Anim Behav. 2014;91:137–50. doi: 10.1016/j.anbehav.2014.03.011

[11] Bräuer J, Call J, Tomasello M. All great ape species follow gaze to distant locations and around barriers. J Comp Psychol. 2005;119(2):145–54. doi: 10.1037/0735-7036.119.2.145 15982158 10.1037/0735-7036.119.2.145

[12] Hattori Y, Kano F, Tomonaga M. Differential sensitivity to conspecific and allospecific cues in chimpanzees and humans: A comparative eye-tracking study. Biology Lett. 2010;6(5):610–3. doi: 10.1098/rsbl.2010.0120 20335197 10.1098/rsbl.2010.0120 PMC2936142

[13] Maranesi M, Ugolotti Serventi F, Bruni S, Bimbi M, Fogassi L, Bonini L. Monkey gaze behaviour during action observation and its relationship to mirror neuron activity. Eur J Neurosci. 2013;38(12):3721–30. doi: 10.1111/ejn.12376 24118599 10.1111/ejn.12376

[14] Kano F, Call J. Great apes generate goal-based action predictions: An eye-tracking study. Psychol Sci. 2014;25(9):1691–8. doi: 10.1177/0956797614536402 25022278 10.1177/0956797614536402

[15] Myowa-Yamakoshi M, Scola C, Hirata S. Humans and chimpanzees attend differently to goal-directed actions. Nat Commun. 2012;3:693 doi: 10.1038/ncomms1695 22353723 10.1038/ncomms1695

[16] Ghazanfar AA, Santos LR. Primate brains in the wild: the sensory bases for social interactions. Nat Rev Neurosci. 2004;5(8):603–16. doi: 10.1038/nrn1473 15263891 10.1038/nrn1473

[17] Senju A, Johnson MH. The eye contact effect: Mechanisms and development. Trends Cogn Sci. 2009;13(3):127–34. doi: 10.1016/j.tics.2008.11.009 19217822 10.1016/j.tics.2008.11.009

[18] Farroni T, Csibra G, Simion F, Johnson MH. Eye contact detection in humans from birth. Proc Nat Acad Sci. 2002;99(14):9602 doi: 10.1073/pnas.152159999 12082186 10.1073/pnas.152159999 PMC123187

[19] Myowa-Yamakoshi M, Tomonaga M, Tanaka M, Matsuzawa T. Preference for human direct gaze in infant chimpanzees (Pan troglodytes). Cognition. 2003;89(2):113–24. doi: 10.1016/S0010-0277(03)00071-4 10.1016/s0010-0277(03)00071-4 12915297

[20] Carpenter M, Nagell K, Tomasello M, Butterworth G, Moore C. Social cognition, joint attention, and communicative competence from 9 to 15 months of age. Monogr Soc Res Child. 1998;63(4):1–174. doi: 10.2307/1166214 9835078

[21] Frank MC, Vul E, Saxe R. Measuring the development of social attention using free-viewing. Infancy. 2012;17(4):355–75. doi: 10.1111/j.1532-7078.2011.00086.x 10.1111/j.1532-7078.2011.00086.x 32693486

[22] Gredebäck G, Fikke L, Melinder A. The development of joint visual attention: a longitudinal study of gaze following during interactions with mothers and strangers. Dev Sci. 2010;13(6):839–48. doi: 10.1111/j.1467-7687.2009.00945.x 20977555 10.1111/j.1467-7687.2009.00945.x

[23] Okamoto S, Tomonaga M, Ishii K, Kawai N, Tanaka M, Matsuzawa T. An infant chimpanzee (Pan troglodytes) follows human gaze. Anim Cogn. 2002;5(2):107–14. 12150035 10.1007/s10071-002-0133-z

[24] Klin A, Jones W, Schultz R, Volkmar F, Cohen D. Visual fixation patterns during viewing of naturalistic social situations as predictors of social competence in individuals with autism. Arch Gen Psychiatry. 2002;59(9):809–16. doi: 10.1001/archpsyc.59.9.809 12215080 10.1001/archpsyc.59.9.809

[25] Dawson G, Toth K, Abbott R, Osterling J, Munson J, Estes A, et al Early social attention impairments in autism: Social orienting, joint attention, and attention to distress. Dev Psychol. 2004;40(2):271–82. doi: 10.1037/0012-1649.40.2.271 14979766 10.1037/0012-1649.40.2.271

[26] Jones W, Klin A. Attention to eyes is present but in decline in 2-6-month-old infants later diagnosed with autism. Nature. 2013;504:427–31. doi: 10.1038/nature12715 24196715 10.1038/nature12715 PMC4035120

[27] Constantino JN, Kennon-McGill S, Weichselbaum C, Marrus N, Haider A, Glowinski AL, et al Infant viewing of social scenes is under genetic control and is atypical in autism. Nature. 2017;in press. doi: 10.1038/nature22999 28700580 10.1038/nature22999 PMC5842695

[28] Lutchmaya S, Baron-Cohen S, Raggatt P. Foetal testosterone and eye contact in 12-month-old human infants. Infant Behav Dev. 2002;25(3):327–35. doi: 10.1016/S0163-6383(02)00094-2

[29] Ebitz RB, Watson KK, Platt ML. Oxytocin blunts social vigilance in the rhesus macaque. Proc Nat Acad Sci. 2013;110(28):11630–5. doi: 10.1073/pnas.1305230110 23798448 10.1073/pnas.1305230110 PMC3710816

[30] Guastella AJ, Mitchell PB, Dadds MR. Oxytocin increases gaze to the eye region of human faces. Biol Psychiatry. 2008;63(1):3–5. doi: 10.1016/j.biopsych.2007.06.026 17888410 10.1016/j.biopsych.2007.06.026

[31] Senju A, Tucker L, Pasco G, Hudry K, Elsabbagh M, Charman T, et al The importance of the eyes: communication skills in infants of blind parents. Proc Roy Soc B. 2013;280:20130436 doi: 10.1098/rspb.2013.0436 23576790 10.1098/rspb.2013.0436 PMC3652463

[32] Senju A, Vernetti A, Ganea N, Hudry K, Tucker L, Charman T, et al Early social experience affects the development of eye gaze processing. Curr Biol. 2015;25(23):3086–91. doi: 10.1016/j.cub.2015.10.019 26752077 10.1016/j.cub.2015.10.019 PMC4683081

[33] Carpenter M, Tomasello M. Joint attention and imitative learning in children, chimpanzees, and enculturated chimpanzees. Soc Dev. 1995;4(3):217–37. doi: 10.1111/j.1467-9507.1995.tb00063.x

[34] Call J, Tomasello M. Production and comprehension of referential pointing by orangutans (Pongo pygmaeus). J Comp Psychol. 1994;108(4):307 doi: 10.1037//0735-7036.108.4.307 7813191 10.1037/0735-7036.108.4.307

[35] Lyn H, Russell JL, Hopkins WD. The impact of environment on the comprehension of declarative communication in apes. Psychol Sci. 2010;21(3):360–5. doi: 10.1177/0956797610362218 20424069 10.1177/0956797610362218 PMC6348075

[36] Blais C, Jack RE, Scheepers C, Fiset D, Caldara R. Culture shapes how we look at faces. PLOS ONE. 2008;3(8):e3022 doi: 10.1371/journal.pone.0003022 18714387 10.1371/journal.pone.0003022 PMC2515341

[37] Kelly DJ, Miellet S, Caldara R. Culture shapes eye movements for visually homogeneous objects. Frontiers in psychology. 2010;1:6 doi: 10.3389/fpsyg.2010.00006 21833189 10.3389/fpsyg.2010.00006 PMC3153738

[38] Gegenfurtner A, Lehtinen E, Säljö R. Expertise differences in the comprehension of visualizations: A meta-analysis of eye-tracking research in professional domains. Educational Psychology Review. 2011;23(4):523–52. doi: 10.1007/s10648-011-9174-7

[39] Vogt S, Magnussen S. Expertise in pictorial perception: eye-movement patterns and visual memory in artists and laymen. Perception. 2007;36(1):91–100. doi: 10.1068/p5262 17357707 10.1068/p5262

[40] Reingold EM, Charness N, Pomplun M, Stampe DM. Visual span in expert chess players: Evidence from eye movements. Psychol Sci. 2001;12(1):48–55. doi: 10.1111/1467-9280.00309 11294228 10.1111/1467-9280.00309

[41] Berg DJ, Boehnke SE, Marino RA, Munoz DP, Itti L. Free viewing of dynamic stimuli by humans and monkeys. J Vis. 2009;9(5):1–15. doi: 10.1167/9.5.19 19757897 10.1167/9.5.19

[42] Einhäuser W, Kruse W, Hoffmann K-P, König P. Differences of monkey and human overt attention under natural conditions. Vision Res. 2006;46(8):1194–209. doi: 10.1016/j.visres.2005.08.032 16375943 10.1016/j.visres.2005.08.032

[43] Kano F, Tomonaga M. Face scanning in chimpanzees and humans: Continuity and discontinuity. Anim Behav. 2010;79(1):227–35. doi: 10.1016/j.anbehav.2009.11.003

[44] Kano F, Call J, Tomonaga M. Face and eye scanning in gorillas, orangutans, and humans: Unique eye-viewing patterns in humans among hominids. J Comp Psychol. 2012;126(4):388–98. doi: 10.1037/a0029615 22946925 10.1037/a0029615

[45] Ghazanfar AA, Nielsen K, Logothetis NK. Eye movements of monkey observers viewing vocalizing conspecifics. Cognition. 2006;101(3):515–29. doi: 10.1016/j.cognition.2005.12.007 16448641 10.1016/j.cognition.2005.12.007

[46] Gothard KM, Erickson CA, Amaral DG. How do rhesus monkeys (Macaca mulatta) scan faces in a visual paired comparison task? Anim Cogn. 2004;7(1):25–36. doi: 10.1007/s10071-003-0179-6 14745584 10.1007/s10071-003-0179-6

[47] Nahm FKD, Perret A, Amaral DG, Albright TD. How do monkeys look at faces? J Cogn Neurosci. 1997;9(5):611–23. doi: 10.1162/jocn.1997.9.5.611 23965120 10.1162/jocn.1997.9.5.611

[48] Keating CF, Keating EG. Visual scan patterns of rhesus monkeys viewing faces. Perception. 1982;11(2):211–9. doi: 10.1068/p110211 7155774 10.1068/p110211

[49] Kano F, Hirata S. Great apes make anticipatory looks based on long-term memory of single events. Curr Biol. 2015;25(19):2513–7. doi: 10.1016/j.cub.2015.08.004 26387711 10.1016/j.cub.2015.08.004

[50] Rochat MJ, Serra E, Fadiga L, Gallese V. The evolution of social cognition: goal familiarity shapes monkeys' action understanding. Curr Biol. 2008;18(3):227–32. doi: 10.1016/j.cub.2007.12.021 18221878 10.1016/j.cub.2007.12.021

[51] Kano F, Hirata S, Call J, Tomonaga M. The visual strategy specific to humans among hominids: A study using the gap-overlap paradigm. Vision Res. 2011;51(23):2348–55. 21951519 10.1016/j.visres.2011.09.006

[52] Hirata S, Fuwa K, Sugama K, Kusunoki K, Fujita S. Facial perception of conspecifics: Chimpanzees (Pan troglodytes) preferentially attend to proper orientation and open eyes. Anim Cogn. 2010;13(5):679–88. doi: 10.1007/s10071-010-0316-y 20213188 10.1007/s10071-010-0316-y

[53] Mundry R, Sommer C. Discriminant function analysis with nonindependent data: consequences and an alternative. Anim Behav. 2007;74(4):965–76. doi: 10.1016/j.anbehav.2006.12.028

[54] Tatler BW. The central fixation bias in scene viewing: Selecting an optimal viewing position independently of motor biases and image feature distributions. J Vis. 2007;7(4). doi: 10.1167/7.14.4 18217799 10.1167/7.14.4

[55] Borg I, Groenen PJ. Modern multidimensional scaling: Theory and applications. 2nd ed: Springer; 2005.

[56] Freeman HD, Ross SR. The impact of atypical early histories on pet or performer chimpanzees. PeerJ. 2014;2:e579 doi: 10.7717/peerj.579 25279262 10.7717/peerj.579 PMC4179557

[57] Freeman HD, Weiss A, Ross SR. Atypical early histories predict lower extraversion in captive chimpanzees. Dev Psychobiol. 2016;58(4):519–27. doi: 10.1002/dev.21395 26814701 10.1002/dev.21395

[58] Kalcher E, Franz C, Crailsheim K, Preuschoft S. Differential onset of infantile deprivation produces distinctive long term effects in adult exlaboratory chimpanzees (Pan troglodytes). Dev Psychobiol. 2008;50(8):777–88. doi: 10.1002/dev.20330 18688804 10.1002/dev.20330

[59] Bloomsmith MA, Baker KC, Ross SR, Lambeth SP. Early rearing conditions and captive chimpanzee behavior: Some surprising findings Nursery rearing of nonhuman primates in the 21st century: Springer; 2006 p. 289–312.

[60] Tomasello M. Joint attention as social cognition Joint attention: Its origins and role in development. Mahwah: Lawrence Erlbaum Assocates; 1995 p. 103–30.

[61] Ghazanfar AA, Shepherd SV. Monkeys at the movies: what evolutionary cinematics tells us about film. Projections. 2011;5(2):1–25. doi: 10.3167/proj.2011.050202

[62] Furuichi T, Sanz C, Koops K, Sakamaki T, Ryu H, Tokuyama N, et al Why do wild bonobos not use tools like chimpanzees do? Behaviour. 2014;152:3–4. doi: 10.1163/1568539X-00003226

[63] van Schaik CP, Deaner RO, Merrill MY. The conditions for tool use in primates: implications for the evolution of material culture. J Hum Evol. 1999;36(6):719–41. doi: 10.1006/jhev.1999.0304 10330335 10.1006/jhev.1999.0304

[64] Call J. Bonobos, chimpanzees and tools: Integrating species-specific psychological biases and socio-ecology In: Hare B, Yamamoto S, editors. Bonobos: Unique in mind, brain, and behavior. New York: Oxford; 2017.

[65] Gruber T, Clay Z, Zuberbühler K. A comparison of bonobo and chimpanzee tool use: evidence for a female bias in the Pan lineage. Anim Behav. 2010;80(6):1023–33. doi: 10.1016/j.anbehav.2010.09.005

[66] Toth N, Schick KD, Savage-Rumbaugh ES, Sevcik RA, Rumbaugh DM. Pan the tool-maker: investigations into the stone tool-making and tool-using capabilities of a bonobo (Pan paniscus). Journal of Archaeological Science. 1993;20(1):81–91. doi: 10.1006/jasc.1993.1006

[67] Koops K, Furuichi T, Hashimoto C. Chimpanzees and bonobos differ in intrinsic motivation for tool use. Scientific reports. 2015;5:11356 doi: 10.1038/srep11356 26079292 10.1038/srep11356 PMC4468814

[68] Herrmann E, Hare B, Call J, Tomasello M. Differences in the cognitive skills of bonobos and chimpanzees. PLOS ONE. 2010;5(8):e12438 doi: 10.1371/journal.pone.0012438 20806062 10.1371/journal.pone.0012438 PMC2929188

[69] Krupenye C, Kano F, Hirata S, Call J, Tomasello M. Great apes anticipate that other individuals will act according to false beliefs. Science. 2016;354(6308):110–4. doi: 10.1126/science.aaf8110 27846501 10.1126/science.aaf8110

[70] van De Waal E, Renevey N, Favre CM, Bshary R. Selective attention to philopatric models causes directed social learning in wild vervet monkeys. Proc Roy Soc B. 2010;277(1691):2105–11. doi: 10.1098/rspb.2009.2260 20236972 10.1098/rspb.2009.2260 PMC2880145

[71] Rizzolatti G, Fogassi L, Gallese V. Neurophysiological mechanisms underlying the understanding and imitation of action. Nat Rev Neurosci. 2001;2(9):661–70. doi: 10.1038/35090060 11533734 10.1038/35090060

[72] Diamond R, Carey S. Why faces are and are not special: An effect of expertise. J Exp Psychol Gen. 1986;115(2):107–17. doi: 10.1037/0096-3445.115.2.107 2940312 10.1037//0096-3445.115.2.107

[73] Kleinke CL. Gaze and eye contact: A research review. Psychol Bullet. 1986;100(1):78–100. doi: 10.1037/0033-2909.100.1.78 3526377

[74] De Waal FB. The communicative repertoire of captive bonobos (*Pan paniscus*), compared to that of chimpanzees. Behaviour. 1988;106(3):183–251. doi: 10.1163/156853988X00269

[75] Rilling JK, Scholz J, Preuss TM, Glasser MF, Errangi BK, Behrens TE. Differences between chimpanzees and bonobos in neural systems supporting social cognition. Social cognitive and affective neuroscience. 2012;7(4):369–79. doi: 10.1093/scan/nsr017 21467047 10.1093/scan/nsr017 PMC3324566

[76] McIntyre MH, Herrmann E, Wobber V, Halbwax M, Mohamba C, de Sousa N, et al Bonobos have a more human-like second-to-fourth finger length ratio (2D: 4D) than chimpanzees: a hypothesized indication of lower prenatal androgens. J Hum Evol. 2009;56(4):361–5. doi: 10.1016/j.jhevol.2008.12.004 19285708 10.1016/j.jhevol.2008.12.004

[77] Sannen A, Heistermann M, Van Elsacker L, Mohle U, Eens M. Urinary testosterone metabolite levels in bonobos: a comparison with chimpanzees in relation to social system. Behaviour. 2003;140(5):683.

[78] Staes N, Stevens JM, Helsen P, Hillyer M, Korody M, Eens M. Oxytocin and vasopressin receptor gene variation as a proximate base for inter-and intraspecific behavioral differences in bonobos and chimpanzees. PLOS ONE. 2014;9(11):e113364 doi: 10.1371/journal.pone.0113364 25405348 10.1371/journal.pone.0113364 PMC4236177

[79] Yamagiwa J. Functioinal analysis of social staring behavior in an all-male group of mountain gorillas. Primates. 1992;33(4):523–44. doi: 10.1007/BF02381153

[80] Thierry B. Unity in diversity: lessons from macaque societies. Evolutionary Anthropology: Issues, News, and Reviews. 2007;16(6):224–38. doi: 10.1002/evan.20147

[81] Thomsen CE. Eye contact by non-human primates toward a human observer. Anim Behav. 1974;22:144–9. doi: 10.1016/S0003-3472(74)80063-1

[82] Gomez JC. Ostensive behavior in great apes: The role of eye contact In: Russon AE, Bard KA, Parker ST, editors. Reaching into thought: The minds of the great apes. New York: Cambridge University Press; 1996 p. 131–51.

[83] Ferrari PF, Paukner A, Ionica C, Suomi SJ. Reciprocal face-to-face communication between rhesus macaque mothers and their newborn infants. Curr Biol. 2009;19(20):1768–72. doi: 10.1016/j.cub.2009.08.055 19818617 10.1016/j.cub.2009.08.055 PMC2784245

