# Supplementary material for: Primate social attention: Species differences and effects of individual experience in humans, great apes, and macaques
Source: PLoS One. 2018 Feb 23;13(2):e0193283. doi: 10.1371/journal.pone.0193283 (PMC5825077; doi:10.1371/journal.pone.0193283)
Supplement: S1 File — (DOCX) [file pone.0193283.s001.docx]

Supplemental Materials

Table A. Participant information in Study 1. Also shown is the percentage of misclassification by pDFA for each participant. (“Nursery/Peer”: reared by human caregivers and conspecific peers; “Mother”: reared by their biological mothers).

| Subject | Species | Facility | Age class | Sex/Gender | Rearing history | % incorrectly classified |
| --- | --- | --- | --- | --- | --- | --- |
| Fimi | Bonobo | WKPRC | Juvenile | Female | Mother | 0 |
| Gemena | Bonobo | WKPRC | Adult | Female | Mother | 21 |
| Ikela | Bonobo | KS | Adult | Female | Nursery/Peer | 55 |
| Jasongo | Bonobo | WKPRC | Adult | Male | Mother | 17 |
| Junior | Bonobo | KS | Adult | Male | Mother | 0 |
| Kuno | Bonobo | WKPRC | Adult | Male | Nursery/Peer | 57 |
| Lenore | Bonobo | KS | Adult | Female | Mother | 0 |
| Lolita | Bonobo | KS | Adult | Female | Nursery/Peer | 0 |
| Louise | Bonobo | KS | Adult | Female | Nursery/Peer | 0 |
| Luiza | Bonobo | WKPRC | Adult | Female | Mother | 11 |
| Vijay | Bonobo | KS | Adult | Male | Nursery/Peer | 2 |
| Yasa | Bonobo | WKPRC | Adult | Female | Mother | 0 |
| Alex | Chimp | WKPRC | Adult | Male | Nursery/Peer | 0 |
| Bangolo | Chimp | WKPRC | Juvenile | Male | Mother | 2 |
| Brigitta | Chimp | WKPRC | Adult | Female | Mother | 35 |
| Daza | Chimp | WKPRC | Adult | Female | Mother | 16 |
| Federike | Chimp | WKPRC | Adult | Female | Mother | 2 |
| Fraukje | Chimp | WKPRC | Adult | Female | Nursery/Peer | 23 |
| Hatsuka | Chimp | KS | Juvenile | Female | Nursery/Peer | 10 |
| Iroha | Chimp | KS | Juvenile | Female | Mother | 10 |
| Jahaga | Chimp | WKPRC | Adult | Female | Mother | 8 |
| Zamba | Chimp | KS | Adult | Male | Mother | 33 |
| Jeudi | Chimp | WKPRC | Adult | Female | Mother | 1 |
| Kara | Chimp | WKPRC | Adult | Female | Mother | 2 |
| Kofi | Chimp | WKPRC | Adult | Male | Mother | 0 |
| Lobo | Chimp | WKPRC | Adult | Male | Mother | 0 |
| Lome | Chimp | WKPRC | Adult | Male | Mother | 5 |
| Misaki | Chimp | KS | Adult | Female | Mother | 12 |
| Mizuki | Chimp | KS | Adult | Female | Nursery/Peer | 20 |
| Natsuki | Chimp | KS | Adult | Female | Mother | 29 |
| Riet | Chimp | WKPRC | Adult | Female | Nursery/Peer | 9 |
| Robert | Chimp | WKPRC | Adult | Male | Mother | 1 |
| Sandra | Chimp | WKPRC | Adult | Female | Mother | 3 |
| Batak | Orangutan | WKPRC | Adult | Male | Mother | 36 |
| Dokana | Orangutan | WKPRC | Adult | Female | Mother | 96 |
| Padana | Orangutan | WKPRC | Adult | Female | Mother | 8 |
| Pini | Orangutan | WKPRC | Adult | Female | Nursery/Peer | 85 |
| Raja | Orangutan | WKPRC | Adult | Female | Mother | 39 |
| Suaq | Orangutan | WKPRC | Adult | Male | Mother | 26 |
| Tanah | Orangutan | WKPRC | Adult | Male | Mother | 99 |
| H01 | Human | - | Adult | Female | - | 0 |
| H02 | Human | - | Adult | Male | - | 0 |
| H03 | Human | - | Adult | Female | - | 0 |
| H04 | Human | - | Adult | Male | - | 0 |
| H05 | Human | - | Adult | Female | - | 0 |
| H06 | Human | - | Adult | Female | - | 0 |
| H07 | Human | - | Adult | Female | - | 0 |
| H08 | Human | - | Adult | Female | - | 0 |
| H09 | Human | - | Adult | Female | - | 0 |
| H10 | Human | - | Adult | Male | - | 23 |
| H11 | Human | - | Adult | Male | - | 0 |
| H12 | Human | - | Adult | Male | - | 0 |
| Sam | Rhesus | TRU | Adult | Male | Mother | 0 |
| Thor | Rhesus | TRU | Adult | Male | Mother | 0 |
| Michel | Rhesus | TRU | Adult | Male | Mother | 100 |
| Milo | Rhesus | TRU | Adult | Male | Mother | 0 |
| Mo | Rhesus | TRU | Adult | Male | Mother | 23 |
| Otis | Rhesus | TRU | Adult | Male | Mother | 0 |
| Yogi | Rhesus | TRU | Adult | Male | Mother | 0 |

Table B. Percent on-screen viewing time (in Experiment 1 and 2).

|  | Species/ Group | Bonobo movie | Chimp movie | Orangutan movie | Macaque movie | Nonprimate movie |
| --- | --- | --- | --- | --- | --- | --- |
| Exp. 1 | Bonobo | 68 | 59 | 48 | 54 | 50 |
|  | Chimpanzee | 79 | 76 | 70 | 76 | 79 |
|  | Orangutan | 73 | 73 | 72 | 81 | 82 |
|  | Human | 96 | 97 | 96 | 96 | 96 |
|  | Macaque | 74 | 77 | 74 | 81 | 78 |
| Exp. 2 | WKPRC chimpanzee | - | 82 | - | - | - |
|  | KS1 chimpanzee | - | 89 | - | - | - |
|  | KS2 chimpanzee | - | 59 | - | - | - |
|  | Human expert adult | - | 93 | - | - | - |
|  | Human novice adult | - | 94 | - | - | - |
|  | Human preschooler | - | 93 | - | - | - |

Table C. Participant information in Study 2. Also shown is the percentage of misclassification by pDFA for each participant. (“Nursery”: reared by human caregivers; “Nursery/Peer”: reared by human caregivers and conspecific peers; “Mother”: mainly reared by their biological mothers; “Watched the cinema”: whether the participant has seen the cinema from which we took our movie stimuli).

| Subject | Species | Facility | Experience | Age class | Sex/  Gender | Rearing history | Watched the cinema | % incorrectly classified |
| --- | --- | --- | --- | --- | --- | --- | --- | --- |
| Alex | Chimpanzee | WKPRC | - | Adult | Male | Nursery/Peer | - | 0 |
| Bangolo | Chimpanzee | WKPRC | - | Juvenile | Male | Mother | - | 11 |
| Fifi | Chimpanzee | WKPRC | - | Adult | Female | Mother | - | 49 |
| Fraukje | Chimpanzee | WKPRC | - | Adult | Female | Nursery/Peer | - | 0 |
| Jahaga | Chimpanzee | WKPRC | - | Adult | Female | Mother | - | 0 |
| Kara | Chimpanzee | WKPRC | - | Adult | Female | Mother | - | 0 |
| Kofi | Chimpanzee | WKPRC | - | Adult | Male | Mother | - | 0 |
| Lobo | Chimpanzee | WKPRC | - | Adult | Male | Mother | - | 0 |
| Lome | Chimpanzee | WKPRC | - | Adult | Male | Mother | - | 0 |
| Riet | Chimpanzee | WKPRC | - | Adult | Female | Nursery/Peer | - | 0 |
| Robert | Chimpanzee | WKPRC | - | Adult | Male | Mother | - | 0 |
| Sandra | Chimpanzee | WKPRC | - | Adult | Female | Mother | - | 0 |
| Trudia | Chimpanzee | WKPRC | - | Adult | Female | Mother | - | 36 |
| Ulla | Chimpanzee | WKPRC | - | Adult | Female | Nursery/Peer | - | 0 |
| Hatsuka | Chimpanzee | KS1 | - | Juvenile | Female | Nursery/Peer | - | 21 |
| Iroha | Chimpanzee | KS1 | - | Juvenile | Female | Mother | - | 0 |
| Zamba | Chimpanzee | KS1 | - | Adult | Male | Mother | - | 100 |
| Misaki | Chimpanzee | KS1 | - | Adult | Female | Mother | - | 2 |
| Mizuki | Chimpanzee | KS1 | - | Adult | Female | Nursery/Peer | - | 0 |
| Natsuki | Chimpanzee | KS1 | - | Adult | Female | Mother | - | 0 |
| Candy | Chimpanzee | KS2 | - | Adult | Female | Nursery | - | 0 |
| Haruna | Chimpanzee | KS2 | - | Adult | Female | Nursery | - | 0 |
| Nacky | Chimpanzee | KS2 | - | Adult | Female | Nursery | - | 1 |
| Nico | Chimpanzee | KS2 | - | Adult | Female | Nursery | - | 0 |
| Oumu | Chimpanzee | KS2 | - | Adult | Female | Nursery | - | 0 |
| Sachi | Chimpanzee | KS2 | - | Adult | Female | Nursery | - | 66 |
| HE01 | Human | - | Expert | Adult | Female | - | Yes | 11 |
| HE02 | Human | - | Expert | Adult | Female | - | Yes | 0 |
| HE03 | Human | - | Expert | Adult | Male | - | Yes | 0 |
| HE04 | Human | - | Expert | Adult | Female | - | Yes | 14 |
| HE05 | Human | - | Expert | Adult | Female | - | Yes | 0 |
| HE06 | Human | - | Expert | Adult | Female | - | Yes | 0 |
| HE07 | Human | - | Expert | Adult | Female | - | Yes | 16 |
| HE08 | Human | - | Expert | Adult | Male | - | Yes | 4 |
| HE09 | Human | - | Expert | Adult | Female | - | Yes | 1 |
| HE10 | Human | - | Expert | Adult | Female | - | Yes | 21 |
| HE11 | Human | - | Expert | Adult | Female | - | Yes | 0 |
| HE12 | Human | - | Expert | Adult | Male | - | No | 7 |
| HE13 | Human | - | Expert | Adult | Male | - | Yes | 1 |
| HE14 | Human | - | Expert | Adult | Male | - | Yes | 1 |
| HE15 | Human | - | Expert | Adult | Male | - | No | 0 |
| HE16 | Human | - | Expert | Adult | Female | - | No | 1 |
| HE17 | Human | - | Expert | Adult | Male | - | No | 0 |
| HE18 | Human | - | Expert | Adult | Female | - | No | 0 |
| HL01 | Human | - | Novice | Adult | Female | - | Yes | 2 |
| HL02 | Human | - | Novice | Adult | Female | - | No | 8 |
| HL03 | Human | - | Novice | Adult | Male | - | No | 12 |
| HL04 | Human | - | Novice | Adult | Male | - | No | 4 |
| HL05 | Human | - | Novice | Adult | Female | - | No | 1 |
| HL06 | Human | - | Novice | Adult | Male | - | No | 0 |
| HL07 | Human | - | Novice | Adult | Female | - | No | 8 |
| HL08 | Human | - | Novice | Adult | Male | - | No | 0 |
| HL09 | Human | - | Novice | Adult | Male | - | Yes | 11 |
| HL10 | Human | - | Novice | Adult | Male | - | Yes | 18 |
| HL11 | Human | - | Novice | Adult | Male | - | Yes | 11 |
| HL12 | Human | - | Novice | Adult | Male | - | No | 23 |
| HL13 | Human | - | Novice | Adult | Female | - | Yes | 7 |
| HL14 | Human | - | Novice | Adult | Female | - | Yes | 7 |
| HL15 | Human | - | Novice | Adult | Male | - | No | 12 |
| HL16 | Human | - | Novice | Adult | Female | - | Yes | 0 |
| HL17 | Human | - | Novice | Adult | Female | - | Yes | 7 |
| HL18 | Human | - | Novice | Adult | Female | - | Yes | 8 |
| HL19 | Human | - | Novice | Adult | Female | - | Yes | 9 |
| HL20 | Human | - | Novice | Adult | Female | - | No | 0 |
| HP01 | Human | - | Novice | Preschooler | Male | - | No | 0 |
| HP02 | Human | - | Novice | Preschooler | Female | - | No | 3 |
| HP03 | Human | - | Novice | Preschooler | Male | - | No | 0 |
| HP04 | Human | - | Novice | Preschooler | Female | - | No | 0 |
| HP05 | Human | - | Novice | Preschooler | Female | - | No | 0 |
| HP06 | Human | - | Novice | Preschooler | Female | - | No | 2 |
| HP07 | Human | - | Novice | Preschooler | Female | - | No | 1 |
| HP08 | Human | - | Novice | Preschooler | Female | - | No | 9 |
| HP09 | Human | - | Novice | Preschooler | Female | - | No | 0 |
| HP10 | Human | - | Novice | Preschooler | Female | - | No | 0 |
| HP11 | Human | - | Novice | Preschooler | Male | - | No | 0 |
| HP12 | Human | - | Novice | Preschooler | Male | - | No | 1 |
| HP13 | Human | - | Novice | Preschooler | Male | - | No | 10 |
| HP14 | Human | - | Novice | Preschooler | Female | - | No | 2 |
| HP15 | Human | - | Novice | Preschooler | Male | - | No | 7 |
| HP16 | Human | - | Novice | Preschooler | Male | - | No | 0 |
| HP17 | Human | - | Novice | Preschooler | Male | - | No | 3 |
| HP18 | Human | - | Novice | Preschooler | Male | - | No | 13 |
| HP19 | Human | - | Novice | Preschooler | Male | - | No | 1 |
| HP20 | Human | - | Novice | Preschooler | Female | - | No | 1 |

Movie links

For the example frames in which Areas-Of-Interest were defined for the models’ eyes, mouth, head (in body), and action targets in Study 1, see the following link.

<https://youtu.be/fb6N8-olJxk>

For the presented movies with all participants’ gaze superimposed in Study 1, see the following link.

<https://youtu.be/qCK-jI-NDs0>

For the presented movies with all participants’ gaze superimposed in Study 2, see the following link. <https://youtu.be/KfVqWAP-D6Q>


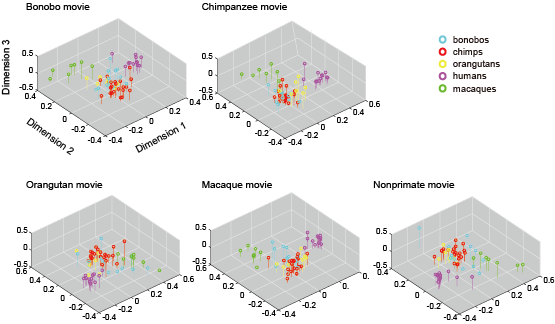


Fig A. Multi-Dimensional Scaling (MDS) maps for movies depicting each species. Note that MDS directions are arbitrary, so maps which can be flipped or rotated to superimpose are equivalent.

**
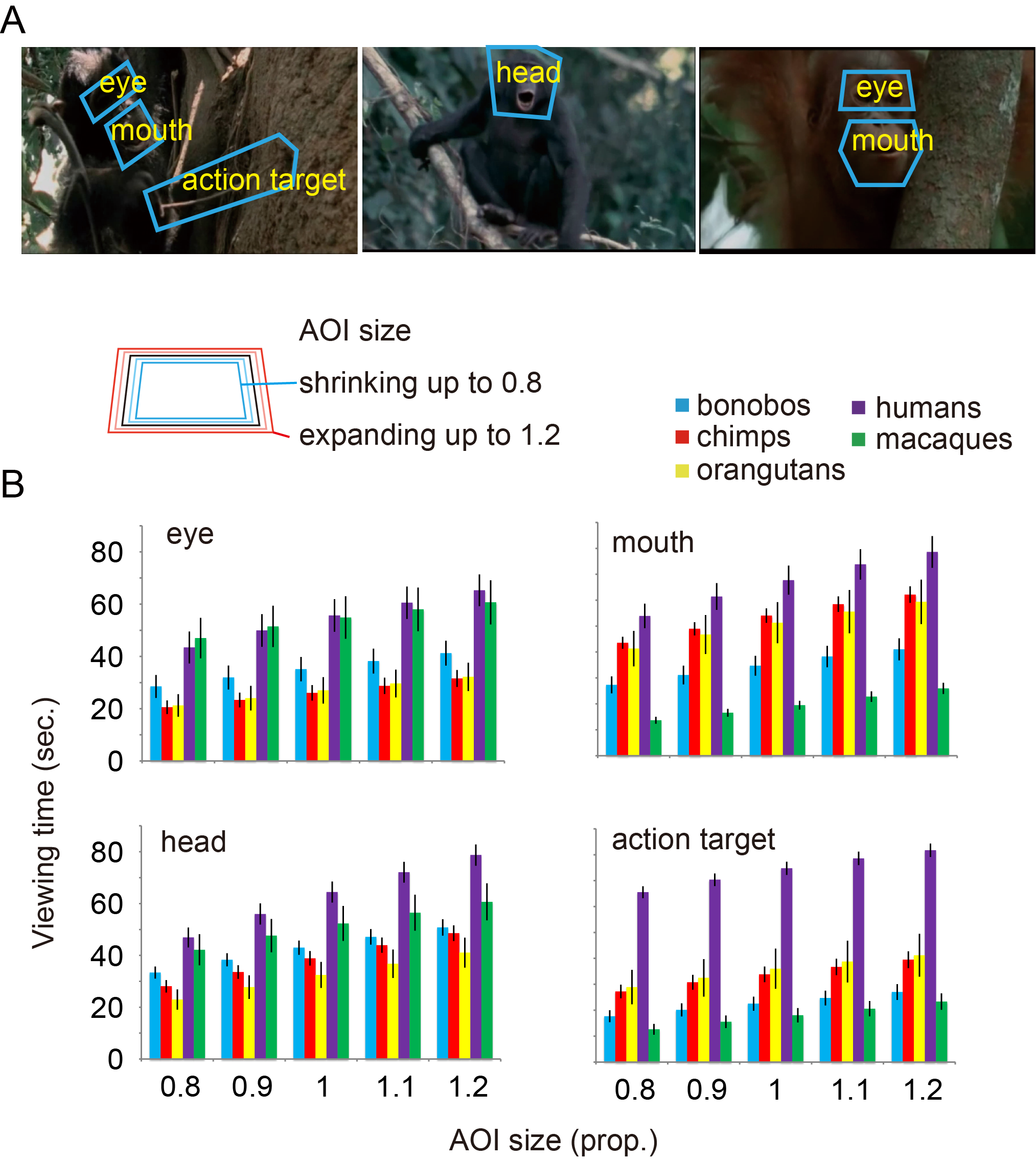
**

Fig B. Results from the control analysis manipulating the size of AOIs. A. Example of defined AOI. B. The total viewing times for AOIs (sec. ± SEM) as a function of the AOI size in Experiment 1. Shrinking or expanding the size of AOI up to 20% did not change the pattern of species differences in these viewing-time data, indicating that the distribution of fixations around each defined AOI was similar across the participant species.

**
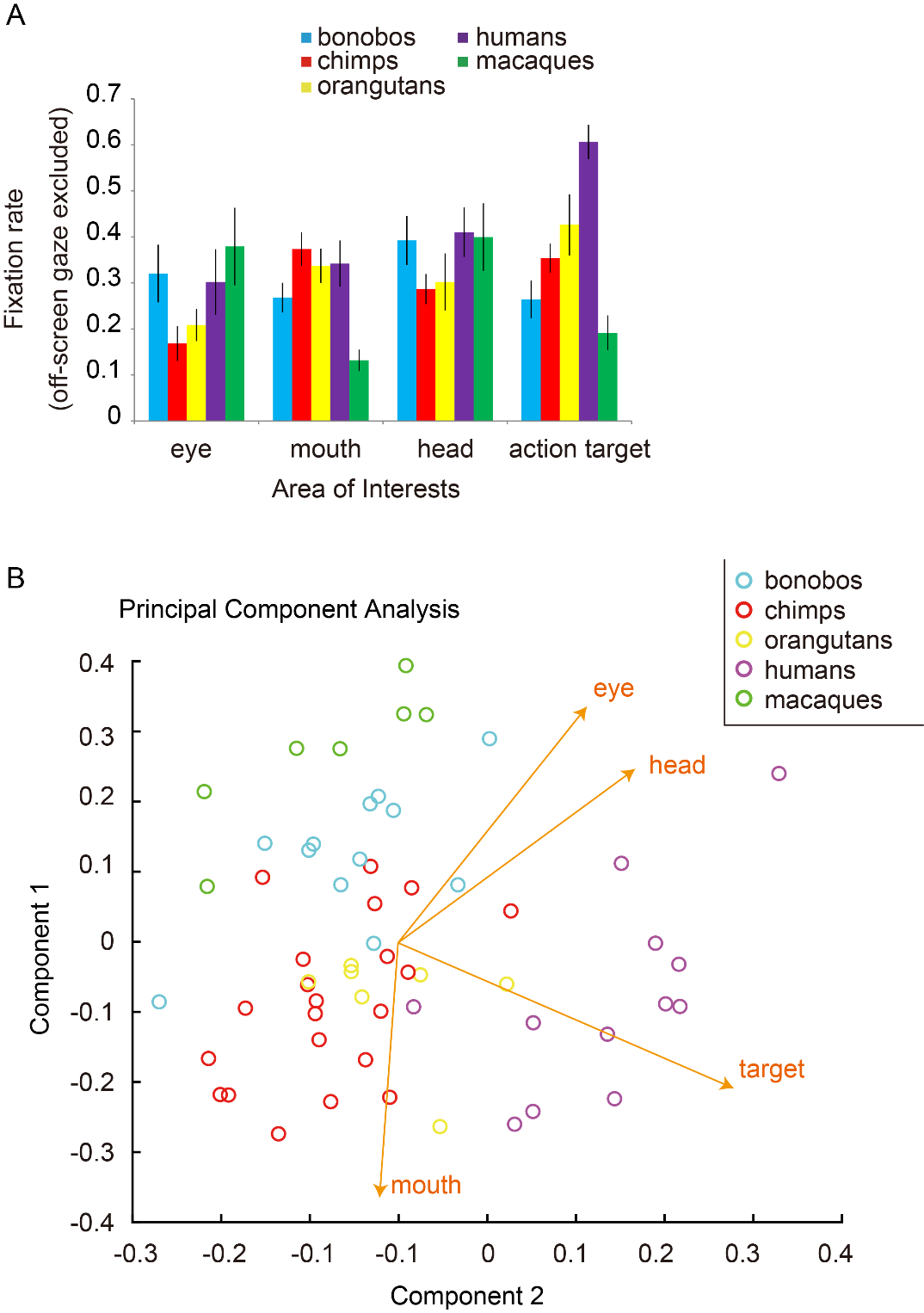
**

Fig C. Replication of the Experiment-1 results (Figure 1) with a different measure, fixation rate. Fixation rate was defined as the number of frames in which they fixate a given AOI (i.e. hit) with respect to the number of frames in which they fixate on-screen while that given AOI was present (i.e. miss).


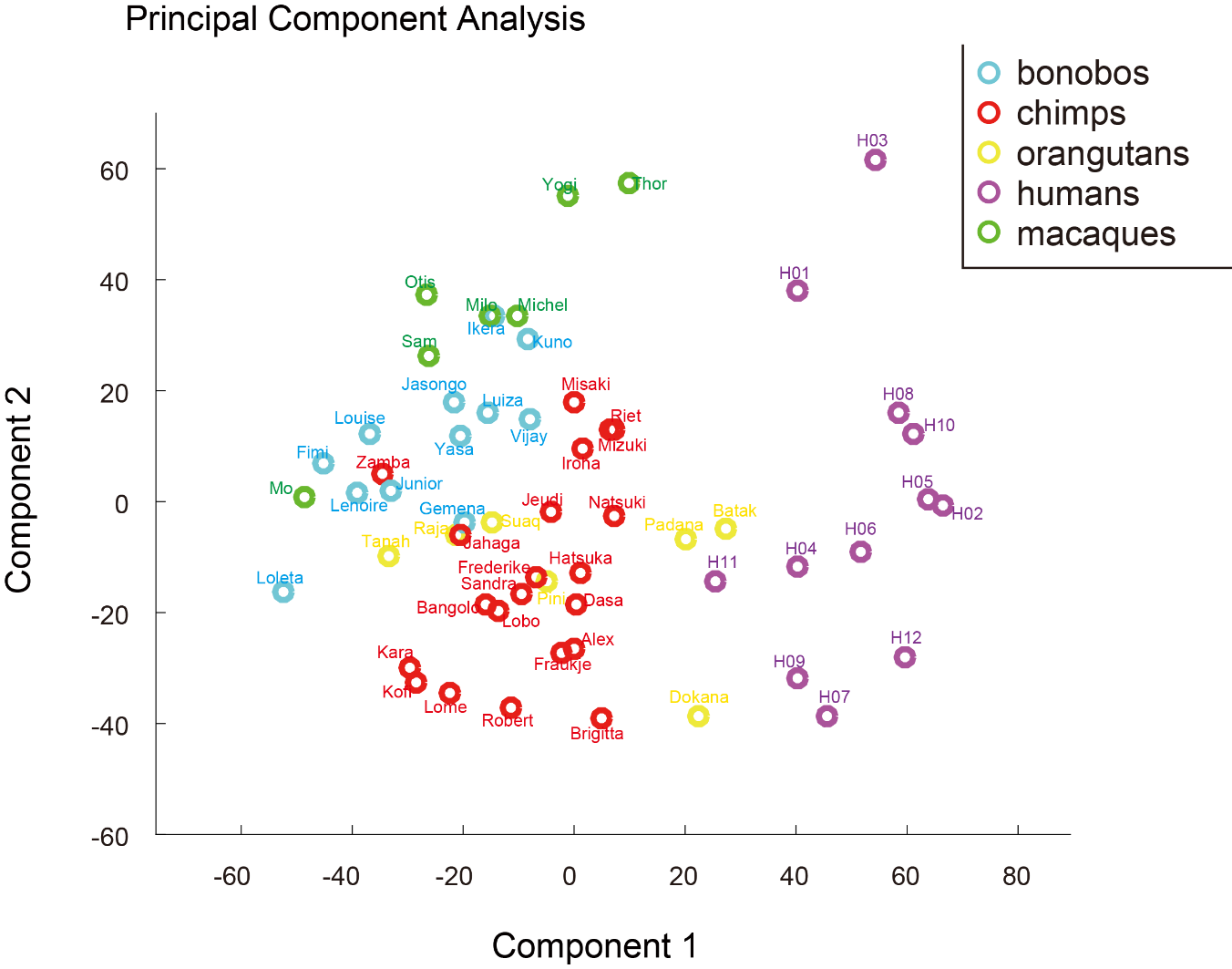


Fig D. Principal Component Analysis (PCA) map based on the viewing times for AOIs in Study 1, with participant names superimposed.


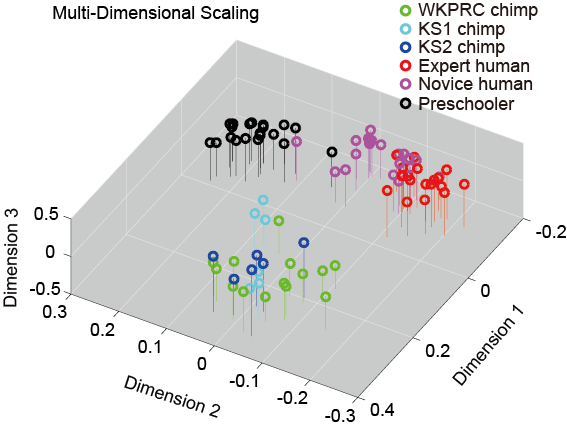


Fig E. MDS map based on Inter-Individual (gaze) Distances (IIDs) in Study 2.


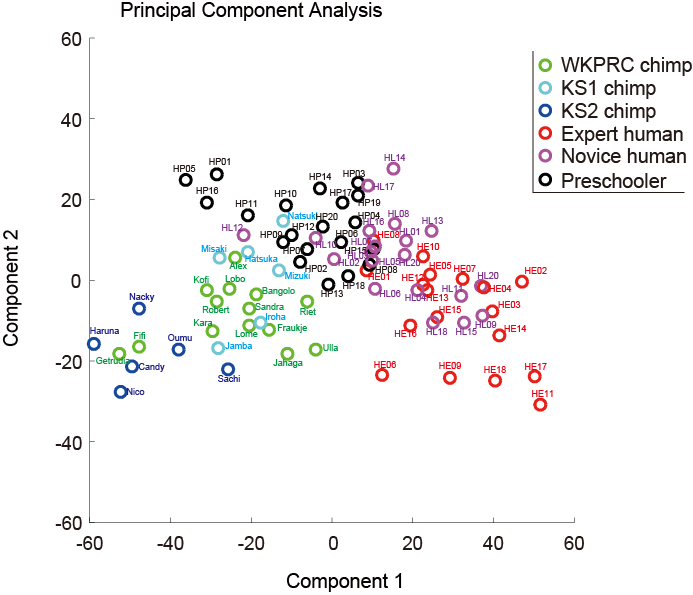


Fig F. PCA map based on the viewing times for AOIs in Study 2, with participant names superimposed.
